# Synthesis and validation of [^18^F]mBPET-1, a fluorine-18 labelled mTOR inhibitor derivative based on a benzofuran backbone

**DOI:** 10.1186/s41181-020-0089-9

**Published:** 2020-01-23

**Authors:** Christian W. Wichmann, Yit Wooi Goh, Adam C. Parslow, Angela Rigopoulos, Nancy Guo, Andrew M. Scott, Uwe Ackermann, Jonathan M. White

**Affiliations:** 1 The University of Melbourne, Parkville, VIC 3010 Australia; 2 Department of Molecular Imaging and Therapy, Austin Hospital, 145 Studley Road, Heidelberg, VIC 3084 Australia; 3 Olivia Newton-John Cancer Research Institute, 145 Studley Road, Heidelberg, VIC 3084 Australia; 4 School of Cancer Medicine, La Trobe University, Plenty Road & Kingsbury Drive, Bundoora, VIC 3086 Australia

**Keywords:** Fluorine-18, ^18^F, mTOR, Everolimus therapy, RAD001, PET, Molecular targeting, Breast cancer

## Abstract

**Background:**

Targeted therapy of HER2 positive breast cancer has led to clinical success in some cases with primary and secondary resistance being major obstacles. Due to the substantial involvement of mTOR kinase in cell growth and proliferation pathways it is now targeted in combination treatments to counteract HER2 targeted therapy resistance. However, the selection of receptive patient populations for a specific drug combination is crucial. This work aims to develop a molecular probe capable of identifying patients with tumour populations which are receptive to RAD001 combination therapy. Based on the structure of a mTOR inhibitor specific for mTORC1, we designed, synthesised and characterised a novel benzofuran based molecular probe which suits late stage fluorination via Click chemistry.

**Results:**

Synthesis of the alkyne precursor 5 proceeded in 27.5% yield over 7 linear steps. Click derivatisation gave the non-radioactive standard in 25% yield. Radiosynthesis of [^18^F]1-((1-(2-Fluoroethyl)-1H-1,2,3-triazol-4-yl) methyl)-4-((5-methoxy-2-phenylbenzofuran-4-yl) methyl) piperazine ([^18^F]mBPET-1) proceeded over two steps which were automated on an iPhase FlexLab synthesis module. In the first step, 2-[^18^F]fluoroethylazide ([^18^F]6) was produced, purified by automated distillation in 60% non-decay-corrected yield and subjected to Click conditions with 5. Semi-preparative RP-HPLC purification and reformulation gave [^18^F]mBPET-1 in 40% ± 5% (*n* = 6) overall RCY with a process time of 90 min. Radiochemical purity was ≥99% at end of synthesis (EOS) and ≥ 98% after 4 h at room temperature. Molar activities ranged from typically 24.8 GBq/μmol (EOS) to a maximum of 78.6 GBq/μmol (EOS). Lipophilicity of [^18^F]mBPET-1 was determined at pH 7.4 (logD_7.4_ = 0.89). [^18^F]mBPET-1 showed high metabolic stability when incubated with mouse S9 liver fractions which resulted in a 0.8% drop in radiochemical purity after 3 h. Cell uptake assays showed 1.3–1.9-fold increased uptake of the [^18^F]mBPET-1 in RAD001 sensitive compared to insensitive cells across a panel of 4 breast cancer cell lines.

**Conclusion:**

Molecular targeting of mTOR with [^18^F]mBPET-1 distinguishes mTOR inhibitor sensitive and insensitive cell lines. Future studies will explore the ability of [^18^F]mBPET-1 to predict response to mTOR inhibitor treatment in in vivo models.

## Introduction

Inhibition of growth signalling receptors such as human epidermal growth factor receptor 2 (HER2) has shown some success in the treatment of breast cancer especially in patients with chemotherapy resistant metastatic disease (Baselga et al. 1996; Cobleigh et al. 1999). However, primary and secondary resistance to HER2 targeted therapies is a common problem among patient populations (Gajria and Chandarlapaty 2011; Narayan et al. 2009). The phosphatidylinositide 3-kinase (PI3K) pathway is a prominent oncogenic signalling pathway downstream of HER2 with the mammalian target of rapamycin (mTOR) as a key mediator (Bjornsti and Houghton 2004). mTOR protein forms a part of two distinct kinases, mTOR complex 1 and 2, which are heavily involved in cell growth and proliferation pathways (Tchevkina and Komelkov 2012). Due to its central role in oncogenesis, mTOR has become a popular target for cancer therapy and a number of mTOR targeted therapeutics have been developed (Liu et al. 2013; Vinayak and Carlson 2013; Wander et al. 2011; Yu et al. 2010; Zhou and Huang 2012). A growing number of preclinical and clinical trials now focus on inhibition of mTOR in combination with drugs targeting other growth signalling pathways such as HER2 (Gayle et al. 2012; Gnant 2013; Hurvitz et al. 2013; Jerusalem et al. 2011; Lu et al. 2007).

Everolimus (RAD001) is an mTOR complex 1 selective kinase inhibitor which has been approved as a chemotherapeutic for a number of indications such as renal cell carcinoma, neuroendocrine tumours and biliary as well as breast cancer (Baselga et al. 2011; Lau et al. 2018; Motzer et al. 2010; Yao et al. 2016).

Due to its wide applicability, RAD001 is gaining popularity as a combination drug. The TRINITI-1 study which examines triplet therapy of hormone receptor positive (HR+), HER2- advanced breast cancer with ribociclib (CDK4/6 inhibition), RAD001 (mTOR inhibition) and exemestane (endocrine therapy) showed clinical benefit in a preliminary analysis in 95 patients (Bardia et al. 2019). Combination of RAD001 with the HER2 targeting monoclonal antibody trastuzumab (Herceptin) is currently being investigated in clinical trials and has progressed to phase 3 (Andre et al. 2010; Hurvitz et al. 2013; Hurvitz et al. 2015a, 2015b; Jerusalem et al. 2011). The BOLERO-3 trial, which focusses on patients with trastuzumab-resistant advanced breast cancer, showed an improvement in median progression-free survival from 5.78 to 7.00 months in the RAD001 cohort compared to the placebo group (Andre et al. 2014). However, closer analysis of biomarker specific response showed that patients with low expression of phosphatase and tensin homolog (PTEN) or high levels of phospho-S6 kinase (pS6) exhibited better response to combination therapy with RAD001. Both low PTEN and high pS6 are indicators of mTOR activation. These results sparked the development of a molecular probe that would allow for the selection of responsive patient populations in a non-invasive manner using positron emission tomography (PET). PET is a non-invasive molecular imaging technique that is capable of visualising biological processes in living organisms. mTOR expression is typically determined via immunohistochemistry (IHC) of tumour biopsies, however these merely provide snapshots of the respective disease and results are highly sampling-dependent (Laes et al. 2017). Molecular imaging techniques can be complementary to biopsy (Adams et al. 2014; Fei and Schuster 2017) or provide advantages especially in the characterisation of deep soft-tissue, lymph node and visceral metastases (Krug et al. 2008).

Currently, there are no mTOR imaging probes for PET or SPECT under investigation in preclinical or clinical studies, neither can a description of the development of such a probe be found in the literature. Very recently, imaging of a related kinase which is part of the same cellular signalling pathway, PI3K, has been described (Altine et al. 2019; Han et al. 2019). In this work, Pictilisib (GDC-0941), a PI3K inhibitor, was radiolabelled with either carbon-11 or fluorine-18 to identify Pictilisib sensitive tumour populations.

**Fig. 1:**
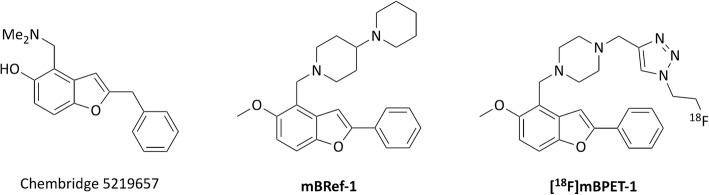
Initial HTS hit Chembridge 5219657, SAR optimized mTOR benzofuran reference compound 1 (mBRef-1) and proposed mTOR benzofuran PET compound 1 ([^18^F]mBPET-1)

In a similar fashion, it was hypothesised that the structure of a mTOR inhibitor could be used as a biological targeting vector to identify cells with high or low RAD001 sensitivity via their mTOR expression or activation levels. The design of the PET ligand was based on work by Salome et al. who have optimized the properties of a novel benzofuran based mTOR inhibitor (Salome et al. 2014a, 2014b). Chembridge 5219657 (Fig. 1) was identified in a high throughput screen (HTS) looking at molecules blocking the nuclear export of Forkhead box protein O1a, which is PI3K/AKT/mTOR pathway dependent (Kau et al. 2003). Salome et al. further characterised this class of compounds via a pull-down assay. Western blot analysis showed that the affigel-coupled derivate exclusively binds to mTOR, or an associated protein, but not to other kinases in the AKT/mTOR pathway. Treatment of SQ20B cancer cells with the benzofuran inhibitor showed dose dependent decrease of pS6 levels and delayed dose dependent increase of pAKT levels. Increased phosphorylation of AKT is a result of mTORC1 inhibition which reduces signalling through a negative feedback loop amplifying PI3K activity which leads to phosphorylation of AKT via PDK1 (Sabatini 2006). These findings were promising since RAD001 is selective for mTORC1.

**Table 1:** Reagent setup to enable automated radiosynthesis of [^18^F]mBPET-1 on iPHASE Flexlab

location	reagent
QMA	Waters QMA Light (activated with 10 mL 0.5 M K_2_CO_3_)
Sep-Pak D	Phenomenex Strata-X C18 (conditioned with EtOH/H_2_O)
vial 1	3.5 mg K_2_CO_3_ in 200 μL H_2_O + 20.0 mg K_222_ in 400 μL MeCN
vial 2	4.0 μL 2-azidoethyl-4-toluenesulfonate in 750 μL MeCN
vial 3	1.0 mL MeCN
vial 7	100 μL DMF
vial 11	10 mL H_2_O
vial 12	1.0 mL DMSO
vial 13	1.0 mL DMSO
vial 17	4 mL H_2_O
vial 18	1.0 mL MeCN
vial 19	6.0 mg Cu (CH_3_CN)_4_ PF_6_ + 4.0 mg TBTA in 300 μL DMF
HPLC-2 flask	40 mL H_2_O
reactor 2	3.0 mg precursor 5 (neat)

Structural variations of Chembridge 5219657 identified the phenyl and the 2-phenoxybenzyl as the most potent substituents in the aryl position (Salome et al. 2014a). Modification of the benzofuran core to an indole, benzoxazole, furopyridine or benzimidazole had adverse effects on the cytotoxicity in SQ20B cancer cells. Modification of the amine substituent in 4-position showed the greatest variation in potency. Replacement of the dimethylamino-group with diethanolamine eliminated the target affinity. Alkylated piperazines were tolerated but surpassed by the 1,4′-bipiperidine and the 4-(dimethylamino)piperidine derivatives which proved to be the most favourable substituents. Attachment of a linker to the bipiperidine allowing for pull-down assays only resulted in a minor loss of potency. Based on these results, the radioligand was designed around a benzofuran core with fluorine-18 attached to a piperazine moiety in 4-position. Copper catalysed click chemistry is a proven method of introducing fluorine-18 in good radiochemical yields (Pretze et al. 2013). A fully automated procedure has been developed which allows convenient derivatisation of alkyne precursors with 2-[^18^F]fluoroethylazide (Ackermann et al. 2014).

In conclusion, the most refined compound to date outperforms the initial structure identified by HTS by about two orders of magnitude and is close to 20-fold more cytotoxic than RAD001. Whether these numbers translate into mTORC1 affinity, however, remains an assumption due to the phenotypic nature of the assay used. Figure 1 shows mTOR benzofuran reference compound 1 (mBRef-1), one of the most potent derivatives of the series (IC50 = 2.5 μM). This structure was altered to give the proposed mTOR benzofuran PET compound 1 ([^18^F]mBPET-1) which suits late-stage fluorination via click chemistry and enables its use in PET.

## Experimental section

### General methods

All chemicals and solvents were purchased from Sigma-Aldrich, Merck, Matrix Scientific, Combi-Blocks and AK Scientific and used as received unless stated otherwise. Analytical thin layer chromatography (TLC) was carried out using aluminium backed 2 mm thick Merck Kieselgel Silica gel 60 GF254 plates. Compounds were visualized under UV_254 nm_ light. Cartridges for solid phase extraction (SPE) of radioactive compounds were purchased from Waters and Phenomenex and conditioned with ethanol and washed with sterile water.

Automated flash chromatography was carried out on a Biotage Isolera One or a GRACE Reveleris system using Biotage SNAP or GRACE Flash cartridges. Semi-preparative reversed-phase high performance liquid chromatography (RP-HPLC) purification of organic molecules was performed on Agilent 1100, 1200 and 1260 series systems equipped with an automated sample injector, diode array UV-detector and automated fraction collector. Mass selective semi-preparative RP-HPLC purification was performed on a Shimadzu LC-20AR HPLC system equipped with a LCMS-2020 module and a FRC-10A fraction collector.

Nuclear magnetic resonance (NMR) spectra for ^1^H, ^13^C and ^19^F nuclei were recorded at ambient temperature using an Agilent MR400 system with autosampler, Varian Unity Inova 500/600 or Bruker Avance III 600 NMR-spectrometers. NMR chemical shifts are reported in parts per million (ppm) and can be followed (in brackets) by multiplicity (s: singlet, d: doublet, t: triplet, m: multiplet, dd: doublet of doublets, dt: doublet of triplets), coupling constants (J) given in Hertz (Hz) and integration.

High-resolution mass spectrometry (HRMS) was performed on a Finnigan LTQ FT hybrid mass spectrometer (Bremen, Germany) linear ion trap with Fourier Transform Ion Cyclotron Resonance (FT-ICR) or on a Thermo Fisher Q Exactive Plus Hybrid Quadrupole-Orbitrap mass spectrometer in conjunction with a Thermo Fisher UltiMate 3000 UHPLC system equipped with an autosampler. Liquid chromatography mass spectrometry (LCMS) was carried out on an Agilent 1200 series RP-HPLC system with an Agilent 6220 Accurate-Mass TOF LC/MS system or on a Shimadzu LC20AD RP-HPLC system with a Shimadzu LCMS-2010 EV mass spectrometer. Gas chromatography-mass spectrometry (GCMS) was performed on an Agilent 7890A GC-system coupled to an Agilent 5975C MSD-module. Mass spectrometric data is expressed as the mass to charge ratio (*m/z*) and is followed by relative intensities with respect to the base peak.

Analytical radio-HPLC was performed on a Shimadzu LC-20 AD gradient HPLC system equipped with a Shimadzu SPD-20A UV/VIS detector and a Bioscan Model 106 radio-HPLC flow-count detector. Semi-preparative RP-HPLC of radiolabelled compounds was performed on the iPHASE FlexLAB associated HPLC system using a Knauer HPLC pump.

Cell lines were obtained from American Type Culture Collection ATCC (Manassas, MD). Cell lines were cultured in RPMI media containing 10% FCS, 1% GlutaMAX (Gibco) and 1% Penicillin Streptomycin (Pen Strep, Gibco). Cells were maintained at 37 °C in humidified 5% CO2 atmosphere. Cell number and viability determination was performed using trypan blue and a Bio-Rad TC20 automated cell counter. Radioactivity was determined using a Perkin Elmer Wizard2 gamma counter. Cell proliferation assays were performed using CellTiter 96® AQueous One Solution (Promega, USA containing 3-(4,5-Dimethylthiazol-2-yl)-5-(3-carboxymethoxyphenyl)-2-(4-sulfophenyl)-2H-tetrazolium (MTS) reagent.

### Chemistry

2-Bromo-1,4-dihydroxybenzene (1). Hydroquinone (10.00 g, 90.8 mmol, 1.00 eq) was dissolved in diethyl ether (100 mL). Bromine (14.51 g, 90.8 mmol, 4.65 mL, 1.00 eq) was added dropwise to the solution under cooling in an ice/water bath. The mixture was stirred at room temperature for 2 h. Subsequently, the mixture was quenched with 10% Na_2_S_2_O_3_ solution (100 mL), extracted with diethyl ether (3 × 100 mL), washed with water (2 × 100 mL) and brine (100 mL), dried over MgSO_4_ and the solvent was removed under vacuum. The product was isolated by automated flash chromatography using an isocratic solvent system and a Grace Reveleris 80 g silica cartridge. Removing the solvent under vacuum gave 1 as a white powder (13.70 g, 80%); Mp: 114.6–116.2 °C (lit. Reference (Akita 1962): 113–115 °C); ^1^H-NMR (500 MHz, DMSO-d_6_) δ 9.34 (s, 1H), 9.03 (s, 1H), 6.85 (d, *J* = 2.9 Hz, 1H), 6.75 (d, *J* = 8.8 Hz, 1H), 6.59 (dd, *J* = 8.8, 2.9 Hz, 1H); ^13^C-NMR (100 MHz, DMSO-d_6_) δ 109.4, 115.8, 117.2, 119.3, 147.0, 150.9; Low-res. EI-MS calculated for C_6_H_5_BrO_2_: 187.95 (100.0%), 189.95 (97.9%), 188.95 (6.6%), 190.95 (6.5%); Found: 187.9 (100.0%), 189.9 (89.1%), 188.9 (9.4%), 190.9 (6.6%).

2-Phenylbenzofuran-5-ol (2). 2-bromo-1,4-dihydroxybenzene (5.00 g, 26.45 mmol, 1.00 eq) and dry triethylamine (5.35 g, 52.91 mmol, 7.40 mL, 2.00 eq) were dissolved in dry THF (32 mL) and acetyl chloride (4.15 g, 52.91 mmol, 3.80 mL, 2.00 eq) was added dropwise at 0 °C. After stirring at room temperature for 5 min diisopropylamine (32 mL), palladium (II) acetate (296 mg, 1.32 mmol, 5 mol%), tri-tert-butylphosphonium tetrafluoroborate (574 mg, 1.98 mmol, 7.5 mol%), copper (I) iodide (251 mg, 1.32 mmol, 5 mol%) and phenylacetylene (5.40 g, 52.91 mmol, 5.80 mL, 2.00 eq) were added to the solution. The reaction was degassed using the freeze-pump-thaw method, heated to 60 °C and stirred for 2 h under nitrogen. Subsequently, methanol (106 mL) and potassium hydroxide (14.80 g, 264.54 mmol, 20.00 eq) in water (21 mL) were added and the solution was stirred at 75 °C for 2 h. After cooling to room temperature the mixture was neutralized with a 10% hydrochloric acid solution and extracted with dichloromethane (3 × 100 mL). The organic phase was washed with brine (150 mL), dried over MgSO_4_ and the solvent was removed under vacuum. Isolation by automated flash chromatography using an isocratic solvent system and a Grace Reveleris 80 g silica cartridge gave 2 as a yellow powder (5.31 g, 96%); Mp: 186.8–188.5 °C (lit. Reference (Alvey et al. 2008; Grinev et al. 1957): 186–186.5 °C or 191 °C); ^1^H-NMR (500 MHz, DMSO-d_6_) δ 9.20 (s, 1H), 7.85 (m, 2H), 7.47 (m, 2H), 7.39 (d, ^3^*J* = 8.8 Hz, 1H), 7.36 (m, 1H), 7.26 (d, ^5^*J* = 0.7 Hz, 1H), 6.93 (d, ^*4*^*J* = 2.4 Hz, 1H), 6.74 (dd, ^*3*^*J* = 8.8 Hz, ^*4*^*J* = 2.7 Hz, 1H); ^13^C-NMR (125 MHz, DMSO-d_6_) δ 156.0, 154.0, 148.9, 130.4, 130.0, 129.4 (2), 129.1, 124.9 (2), 113.8, 111.8, 105.8, 102.4; Low-res. EI-MS calculated for C_14_H_10_O_2_: 210.07 (100.0%), 211.07 (15.3%), 212.07 (1.5%); Found: 210.1 (100%), 211.1 (17.2%), 212.1 (1.8%).

5-hydroxy-2-phenylbenzofuran-4-carbaldehyde (3a). Paraformaldehyde (857 mg, 28.54 mmol, 6.0 eq), magnesium chloride (770 mg, 8.09 mmol, 1.7 eq) and triethylamine (9.51 mmol, 1.33 mL, 2.0 eq) were added to a stirred solution of 2-phenylbenzofuran-5-ol (1.00 g, 4.76 mmol, 1.0 eq) in acetonitrile (25 mL). The mixture was heated to reflux for 19 h and the yellow suspension was subsequently quenched with 10% aq. HCl (10 mL). The aqueous layer was extracted with EtOAc (3 × 30 mL). The combined organic layers were washed with water (50 mL), brine (50 mL), dried over MgSO_4_ and the solvents were evaporated under reduced pressure to give the crude product as a brown solid. The product was isolated by automated flash chromatography to give 3a as bright yellow crystals (741 mg, 65%). Mp: 121.5–122.9 °C; ^1^H-NMR (600 MHz, CDCl_3_) δ 11.40 (s, 1H), 10.35 (d, ^4^*J* = 0.4 Hz, 1H), 7.87 (m, 2H), 7.64 (dd, ^3^*J* = 8.9 Hz, ^5^*J* = 0.7 Hz, 1H), 7.47 (m, 2H), 7.40 (tt, ^3^*J* = 7.4 Hz, ^4^*J* = 1.4 Hz, 1H), 7.30 (d, ^5^*J* = 0.9 Hz, 1H), 6.86 (dd, ^3^*J* = 8.9 Hz, ^4^*J* = 0.4 Hz, 1H); ^13^C-NMR (150 MHz, CDCl_3_) δ 192.6, 159.8, 159.4, 148.8, 130.8, 129.6, 129.4, 128.9 (2), 125.2 (2), 120.2, 113.8, 111.4, 97.6; Low-res. EI-MS calculated for C_15_H_10_O_3_: 238.06 (100.0%), 239.07 (16.5%), 240.07 (1.9%); Found: 238.1 (100.0%), 239.1 (16.4%), 240.1 (1.9%).

5-Methoxy-2-phenylbenzofuran-4-carbaldehyde (3b). Methyl iodide (523 μL, 8.39 mmol, 2.0 eq) and potassium carbonate (1.16 g, 8.39 mmol, 2.0 eq) were added to a solution of 5-methoxy-2-phenylbenzofuran (1.00 g, 4.20 mmol, 1.0 eq) in acetone (20 mL). The mixture was heated in a sealed tube at 55 °C for 18 h. The reaction was then cooled to room temperature and the solvent was removed under a stream of nitrogen. The residue was dissolved in water (50 mL) and extracted with dichloromethane (3 × 50 mL). The combined organic layers were washed with brine (100 mL), dried over MgSO_4_ and the solvent was removed under vacuum which afforded 3b as a yellow powder (1.06 g, 99%). Mp: 106.5–110.3 °C; ^1^H-NMR (600 MHz, CDCl_3_) δ 10.64 (s, 1H), 7.90 (d, *J* = 7.3 Hz, 2H), 7.83 (s, 1H), 7.64 (d, *J* = 8.9 Hz, 1H), 7.45 (t, *J* = 7.6 Hz, 2H), 7.37 (t, *J* = 7.3 Hz, 1H), 6.90 (d, *J* = 8.8 Hz, 1H), 3.96 (s, 3H); ^13^C-NMR (150 MHz, CDCl_3_) δ 190.3, 160.2, 160.0, 150.0, 130.0, 129.3, 129.3, 128.9 (2), 125.4 (2), 117.7, 116.6, 108.0, 102.6, 56.7; Low-res. EI-MS calculated for C_16_H_12_O_3_: 252.08 (100.0%), 253.08 (17.6%), 254.09 (1.4%); Found: 252.1 (100.0%), 253.1 (17.3%), 254.1 (2.0%).

1-(Prop-2-yn-1-yl) piperazine (4). Propargyl bromide (1.19 g, 1.11 mL, 10.0 mmol, 1.00 eq) and piperazine (8.61 g, 100 mmol, 10.00 eq) were dissolved in THF (90 mL) and potassium carbonate (4.15 g, 30.0 mmol, 3.00 eq) was added. The reaction was stirred for 4 h under reflux. Subsequently, the solvent was removed under vacuum and the residue was dissolved in water (40 mL). The aqueous phase was extracted with ethyl acetate (4 × 40 mL), washed with brine (2 × 40 mL), dried over MgSO_4_ and the solvent was removed under vacuum to afford 4 as a light brown powder (174 mg, 14%); ^1^H-NMR (500 MHz, CDCl_3_) δ 3.28 (d, *J* = 2.5 Hz, 2H), 2.93 (t, *J* = 4.9 Hz, 4H), 2.65 (s, 1H), 2.54 (m, 4H), 2.25 (t, *J* = 2.5 Hz, 1H); ^13^C-NMR (125 MHz, CDCl_3_) δ 73.2, 53.1 (2), 51.7, 47.4, 46.0 (2); HRMS calculated for C_7_H_13_N_2_ (MH^+^): 125.10732; Found: 125.10734.

1-((5-Methoxy-2-phenylbenzofuran-4-yl) methyl)-4-(prop-2-yn-1-yl) piperazine (5). Catalytic acetic acid (2 drops), followed by sodium cyanoborohydride (50 mg, 0.79 mmol, 2.0 eq) was added to a stirred solution of 5-methoxy-2-phenylbenzofuran-4-carbaldehyde (100 mg, 0.40 mmol, 1.0 eq) and 1-(prop-2-yn-1-yl) piperazine (49 mg, 0.40 mmol, 1.0 eq) in methanol (10 mL). The mixture was stirred at room temperature for 18 h. The solvent was removed under vacuum and the product was isolated by automated flash chromatography to yield 5 as a yellow oil (78 mg, 55%); ^1^H-NMR (500 MHz, CDCl_3_) δ 7.88 (d, *J* = 7.1 Hz, 2H), 7.46 (t, *J* = 7.7 Hz, 2H), 7.36 (m, 2H), 7.25 (s, 1H), 6.91 (d, *J* = 8.8 Hz, 1H), 3.87 (s, 3H), 3.84 (s, 2H), 3.29 (d, *J* = 2.4 Hz, 2H), 2.62 (br. s., 8H), 2.24 (t, *J* = 2.4 Hz, 1H); ^13^C-NMR (126 MHz, CDCl_3_) δ 156.4, 154.1, 150.0, 130.9, 130.6, 128.7 (2), 128.5, 124.9 (2), 118.1, 110.0, 109.6, 101.4, 79.1, 73.0, 57.3, 53.8, 53.0 (2), 52.1 (2), 46.8; HRMS calculated for C_23_H_25_N_2_O_2_ (MH^+^): 361.19105; Found: 361.18994.

2-Fluoroethylazide (6). Sodium azide (65 mg, 1.00 mmol) was added to a solution of 2-fluoroethyl 4-toluenesulfonate (218 mg, 1.00 mmol) in dimethylformamide (10 mL). The mixture was stirred for 24 h at room temperature and used for subsequent Click Chemistry reactions without further purification.

1-((1-(2-Fluoroethyl)-1H-1,2,3-triazol-4-yl) methyl)-4-((5-methoxy-2-phenylbenzofuran-4-yl) methyl) piperazine (mBPET-1). Copper (I) iodide (2.5 mg, 0.013 mmol, 10 mol%) and sodium ascorbate (26 mg, 0.13 mmol, 1.1 eq) were dissolved in water (250 μL). Subsequently, acetonitrile (250 μL), dimethylformamide (200 μL) and diisopropylethylamine (25 μL, 0.14 mmol, 1.2 eq) were added and mixed thoroughly to give a cloudy orange mixture. Freshly prepared 2-fluoroethylazide solution (1.2 mL, 0.12 mmol, 1.0 eq) was added to a stirred solution of 5 (30 mg, 0.12 mmol, 1.0 eq) in dimethylformamide (300 μL). The catalyst mixture was then added and the reaction was left to stir at room temperature for 2 h. Upon completion the reaction was concentrated under reduced pressure and extracted with ethyl acetate (3 × 3 mL). The combined organic phases were dried over MgSO_4_ and the solvent was removed under reduced pressure to give a yellow oil. mBPET-1 was isolated by semi-preparative RP-HPLC and obtained as its TFA salt in the form of a white powder (20 mg, 25%); ^1^H-NMR (600 MHz, CDCl_3_) δ 7.90 (s, 1H), 7.87 (d, *J* = 7.3 Hz, 2H), 7.56 (d, *J* = 8.8 Hz, 1H), 7.46 (t, *J* = 7.6 Hz, 2H), 7.39 (t, *J* = 7.4 Hz, 1H), 7.21 (s, 1H), 6.91 (d, *J* = 9.0 Hz, 1H), 4.79 (dt, ^2^*J*_HF_ = 46.6 Hz, ^3^*J*_HH_ = 4.7 Hz, 2H), 4.68 (dt, ^3^*J*_HF_ = 26.6 Hz, ^3^*J*_HH_ = 4.7 Hz, 2H), 4.52 (s, 2H), 4.28 (s, 2H), 3.89 (s, 3H), 3.62 (br. s., 4H), 3.56 (br. s., 4H); ^13^C-NMR (151 MHz, CDCl_3_) δ 158.9, 155.0, 149.5, 136.8, 132.2, 129.5, 129.3, 128.8 (2), 126.6, 125.2 (2), 113.9, 107.7, 106.0, 99.1, 81.6, 80.4, 56.2, 52.4 (2), 50.9 (2), 50.7, 48.3 (d, ^1^*J*_CF_ = 25.4 Hz); HRMS calculated for C_25_H_29_FN_5_O_2_ (MH^+^): 450.22998; Found: 450.22839.

1′-((5-methoxy-2-phenylbenzofuran-4-yl) methyl)-1,4′-bipiperidine (mBRef-1). Catalytic acetic acid (2 drops), followed by sodium cyanoborohydride (100 mg, 1.59 mmol, 2.0 eq) was added to a stirred solution of 5-methoxy-2-phenylbenzofuran-4-carbaldehyde (200 mg, 0.79 mmol, 1.0 eq) and 4-piperidinopiperidine (133 mg, 0.79 mmol, 1.0 eq) in methanol (20 mL). The mixture was stirred at room temperature for 48 h. The solvent was removed under vacuum and the product was isolated by automated flash chromatography and RP-HPLC to yield mBRef-1 as a light yellow powder (187 mg, 58%); Mp: 225.3–229.0 °C; ^1^H-NMR (500 MHz, CDCl_3_) δ 7.88 (m, 2H), 7.45 (m, 2H), 7.36 (m, 2H), 7.27 (s, 1H), 6.90 (d, *J* = 8.8 Hz, 1H), 3.87 (s, 3H), 3.80 (s, 2H), 3.03 (d, *J* = 11.7 Hz, 2H), 2.50 (br. s., 4H), 2.22 (m, 1H), 2.07 (td, *J* = 11.9 Hz, *J* = 2.0 Hz, 2H), 1.78 (d, *J* = 12.2 Hz, 2H), 1.58 (br. s., 6H), 1.43 (br. s., 2H); ^13^C-NMR (126 MHz, CDCl_3_) δ 156.3, 154.0, 150.0, 130.9, 130.7, 128.7 (2), 128.4, 124.9 (2), 118.9, 109.7, 109.7, 101.6, 62.9, 57.4, 54.0, 53.6 (2), 50.3 (2), 28.2 (2), 26.4 (2), 24.8; HRMS calculated for C_26_H_32_N_2_O_2_ (MH^+^): 405.25366; Found: 405.25227.

### Radiochemistry

No-carrier-added (n.c.a.) [^18^F]fluoride was produced via the ^18^O (p, n)^18^F nuclear reaction by bombardment of an isotopically enriched [^18^O]-H_2_O target with either a 18 MeV or a 10 MeV proton beam generated by an IBA 18/9 or 10/5 cyclotron at the Austin Hospital (Heidelberg, Australia). [^18^F]Fluoride was concentrated on a Waters Sep-Pak Accell Plus QMA Light cartridge which was conditioned with 10 mL 0.5 M aqueous potassium carbonate solution and washed with 10 mL water prior to use.

[^18^F]1-((1-(2-Fluoroethyl)-1H-1,2,3-triazol-4-yl) methyl)-4-((5-methoxy-2-phenylbenzofuran-4-yl) methyl) piperazine ([^18^F]mBPET-1). [^18^F]Fluoride (3.7–74 GBq) was eluted from the QMA light ion exchange cartridge using a solution of 3.5 mg potassium carbonate in 200 μL water and 20 mg Kryptofix 2.2.2 in 400 μL acetonitrile. The eluate was dried azeotropically at 75 °C under vacuum and a gentle argon stream over 6 min followed by addition of 1 mL acetonitrile and drying at 120 °C under full vacuum for 5 min. 4.0 μL 2-azidoethyl-4-toluenesulfonate in 750 μL acetonitrile were added to the dry ^18^F^−^/cryptate and the reaction was heated to 90 °C for 7 min. [^18^F]6 was distilled into a cooled vessel containing 3.0 mg alkyne-precursor 5. Subsequently a mixture of 6.0 mg tetrakis (acetonitrile) copper (I) hexafluorophosphate and 4.0 mg tris [(1-benzyl-1*H*-1,2,3-triazol-4-yl) methyl] amine (TBTA) in 300 μL DMF was added to the distilled product and heated to 70 °C for 30 min in a sealed reactor. The reaction was heated for another 7 min under vacuum before adding 1 mL acetonitrile and 4 mL water. The diluted mixture was purified on a semi-preparative RP-HPLC column (Phenomenex, Gemini 10u C18, 250 × 10 mm, 10 μm; gradient: 20–90% acetonitrile in 0.1 M aq. ammonium formate over 18 min; flow rate: 4 mL/min). [^18^F]mBPET-1 eluted at 15 min and was diluted with 40 mL of deionized water upon collection, passed through a conditioned Phenomenex Strata-X C18 cartridge, washed with 10 mL of deionized water and reformulated using 2 mL DMSO. The overall radiochemical yield (RCY) of [^18^F]mBPET-1 was 40% ± 5% (*n* = 6) after 90 min. A sample of the reconstituted product was analysed by analytical RP-HPLC (Phenomenex, Gemini 5u C18, 150 × 4.6 mm, 5 μm; gradient: 5–90% acetonitrile in 0.1 M aq. ammonium formate over 18 min; flow rate: 0.5 mL/min, t_R_ = 13.6 min) to determine the molar activity and radiochemical purity.

### Lipophilicity

The lipophilicity of [^18^F]mBPET-1 was determined by addition of 444 kBq to a mixture of 800 μL *n*-octanol and 800 μL 0.1 M phosphate buffer (pH 7.4). The mixture was vortexed for 3 min and centrifuged at 10.000 rpm for 5 min. The radioactivity in 100 μL aliquots of each phase was measured in a gamma counter. Experiments were performed in triplicates.

### Metabolic stability assay

The metabolic stability of [^18^F]mBPET-1 was assessed using mouse S9 liver fractions. NADPH was generated in-situ using a NADPH generating system. To create 800 μL of the NADPH generating stock 200 μL aliquots of the following solutions were mixed: 9.3 mg glucose-6-phosphate in 250 μL PBS; 9.95 mg ß-nicotinamide adenine dinucleotide phosphate sodium salt hydrate (NADP sodium salt hydrate) in 250 μL PBS; 6.7 mg magnesium chloride hexahydrate in 250 μL Milli-Q water; 6 μL (11.5 U) glucose-6-phosphate dehydrogenase (G-6-P-DH) stock solution in 1 mL PBS. The G-6-P-DH stock solution (2000 U/mL) was prepared by dissolving 2.33 mg protein in 1 mL PBS and stored at -30 °C. The assay was carried out by mixing 230 μL of PBS with 40 μL NADPH generating solution, 15 μL S9 liver fraction stock and 15 μL analyte in 10% ethanol/saline containing about 3.7 MBq (100 μCi). The control study was prepared accordingly without the S9 liver fraction stock. The solutions were incubated at 37 °C in a thermo shaker. Samples were quenched with 150 μL methanol at various time points and centrifuged at 12.000 rpm for 10 min. The supernatant was analysed using radio-HPLC.

### MTS proliferation assay

Two thousand five hundred cells per well were seeded in a 96-well plate with column 12 left as a blank and incubated in 50 μL media overnight at 37 °C. Subsequently, 50 μL of media containing different concentrations of RAD001 was added to the wells. Drug media concentrations were prepared by performing 3:4 serial dilutions over 10 wells in triplicates on separate plates. The drug media was then transferred to the cell plates, leaving an “untreated” and a blank well. Cells were incubated for 3 days followed by addition of 20 μL of MTS reagent. Plates were incubated for a further 90–180 min at 37 °C before the absorption at 490 nm was measured on a microplate reader using a 650 nm reference. The average absorbance of the blank wells was subtracted from the absorbances in the untreated and treated wells. The latter were then expressed as percentiles of the average absorbance of untreated wells and normalized. EC50 values were determined using the PRISM software package and a four parameter variable slope non-linear curve fit. Experiments were performed in triplicates and repeated three times. Statistical significance was calculated using a one-way ANOVA comparison with Bonferroni post hoc test (^****^ indicates *p* ≤ 0.0001).

### Cell uptake assay

Four hundred thousand cells per well were seeded in a 6-well plate and incubated in 5 mL media overnight at 37 °C. Subsequently the media was replaced with 2.0 mL media containing 1% FCS and wells were treated in triplicates with 370–555 kBq of [^18^F]mBPET-1 in < 5% DMSO/media (0% FCS). For blocked experiments, cells were additionally treated with mBRef-1 at a concentration of 10 μM. Plates were incubated as per their respective time points (5, 10, 15, 20, 30, 60, 90 and 120 min) and quenched by removal of the media and washing twice with 0.5 mL cold PBS. The supernatant and washing solutions were combined and the radioactivity was measured in a gamma counter (S). Optionally, the second washing step was replaced by an acid wash to remove surface bound [^18^F]mBPET-1 using 0.5 mL of cold 50 mM sodium acetate buffer (pH 5) followed by washing twice with 1 mL cold PBS. The acetate and PBS wash solutions were combined and the radioactivity was counted in a gamma counter (A). The cell pellet was lysed using 0.8 mL of cold 0.1 M sodium hydroxide solution and the lysate was collected. The well was rinsed twice with 1 mL cold PBS and the radioactivity in the combined fractions was determined in a gamma counter (P). Radioactivity readouts were correct for background radiation (*) and the cell uptake fraction of [^18^F]mBPET-1 was calculated as: P* / (S* + A* + P*). The cell surface bound fraction of [^18^F]mBPET-1 was calculated as: A* / (S* + A* + P*). Experiments were performed in triplicates and repeated 1–8 times. Statistical significance was calculated using a non-parametric one-way ANOVA comparison with Dunn’s post hoc testing (^*^ indicates *p* ≤ 0.05,^**^ indicates *p* ≤ 0.01, ^***^ indicates *p* ≤ 0.001, ^****^ indicates *p* ≤ 0.0001).

## Results and discussion

**Fig. 2:**
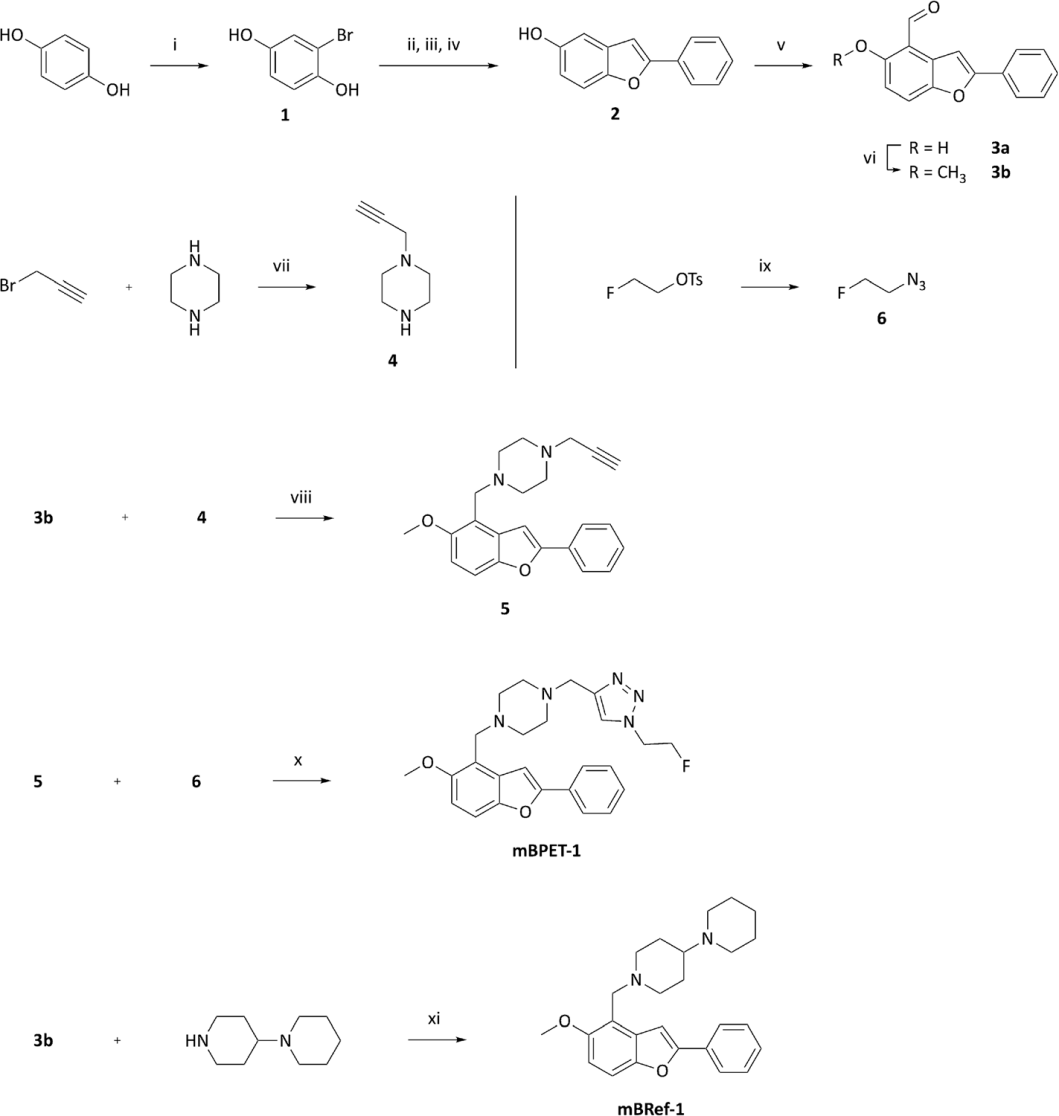
Synthesis of alkyne precursor 5, non-radioactive mBPET-1, and mBRef-1. i) Br_2_, Et_2_O, 2 h, rt., 80%; ii) acetyl chloride, Et_3_N, THF, 5 min, 0 °C; iii) phenylacetylene, 5 mol% Pd (OAc)_2_, 7.5 mol% P^t^Bu_3_*HBF_4_, 5 mol% CuI, diisopropylamine, 2 h, 60 °C; iv) KOH/aq. methanol, 2 h, 75 °C, 96%; v) p-HCHO, Et_3_N, MgCl_2_, acetonitrile, reflux, 19 h, 65%; vi) MeI, K_2_CO_3_, acetone, 18 h, 55 °C, quant.; vii) K_2_CO_3_, THF, reflux, 4 h, 14%; viii) NaBH_3_CN, acetic acid (cat.), methanol, 18 h, rt., 55%; ix) NaN_3_, DMF, 24 h, rt.; x) 10 mol% CuI, sodium ascorbate, diisopropylethylamine, H_2_O/MeCN/DMF, 2 h, rt., 25%; xi) NaBH_3_CN, acetic acid (cat.), methanol, 48 h, rt., 58%

Initially, the synthesis towards mBPET-1 was based closely on results from the structural optimisation work described above. The published reaction sequence relies on a Mannich reaction which could not be reproduced with the building blocks used in this work. Replacement with a sequence of ortho-formylation and subsequent reductive amination allowed access to different amino methylated compounds in reproducible yields (Fig. 2).

In the first step, hydroquinone was brominated using bromine to give the mono-brominated product 1 in 80% yield. Bromination in more than one position was not observed. Bromohydroquinone was then reacted with acetyl chloride to mask the hydroxyl groups as acetoxy groups. The protected compound and phenylacetylene were then subjected to Sonogashira conditions using copper iodide and palladium / tri-*tert*-butylphospine catalyst systems and diisopropylamine as a base. The initial protection step, the cross-coupling reaction as well as the subsequent deprotection and condensation with potassium hydroxide were carried out in a one-pot fashion without intermediate purification. Chromatographic isolation gave the fully formed benzofuran 2 in excellent yields up to 96%. Ring closure was confirmed using DEPT135 and standard ^13^C-NMR spectroscopy. Over the course of the furan ring formation, the number of quaternary carbons in the compound is reduced from 6 to 5 which was confirmed in the recorded spectra. Scale-up did not affect the Sonogashira reaction negatively and yields were consistently high when the reaction mixture was carefully degassed prior to heating the reaction (Elangovan et al. 2003). Oxygen contamination is known to lead to homocoupling between terminal alkynes as described by the Glaser coupling (Glaser 1869; Glaser 1870). The corresponding product, 1,3-butadiyne, was detected in reaction mixtures that showed reduced yields.

A two-step procedure to *ortho*-formylate benzofuran substrates using ethyl *N*-phenylformimidate to form the imine followed by hydrolysis under acidic conditions has been reported (Andrew et al. 1999). A more convenient method published by Hofsløkken and Skattebøl describes the *ortho*-formylation of various phenols in one step (Hofsløkken and Skattebøl 1999). The scope of this reaction, now called Casnati-Skattebøl formylation, has recently been expanded (Akselsen et al. 2009; Hansen and Skattebøl 2005; Hansen and Skattebol 2012; Pergomet et al. 2017). Although this method had not been tested on benzofurans, it was investigated whether 2 could be formylated using the reported conditions. The reaction requires a minimum of 2 equivalents of formaldehyde relative to the amount of phenol. Using 6 equivalents, formation of the aldehyde in one step was achieved in 65% yield. Magnesium chloride which acts as a Lewis acid coordinates to the phenol and formaldehyde, directing it into the least sterically hindered *ortho*-position. Neither the 4- nor the 6-position of 2 are particularly crowded hence regioselectivity was expected to be poor. However, the reaction gave 3a as the major product as confirmed by the presence of vicinal proton coupling in the ^1^H-NMR spectrum between the protons in 6- and 7-position (Additional file 1: Figure S3 a/c).

Preparation of 1-(Prop-2-yn-1-yl) piperazine (4) was attempted at low temperatures following published procedures (Castelhano et al. 2003; Hamann et al. 2014; Kushwaha and Kaushik 2014). However, product could only be isolated when the reaction was performed at elevated temperatures in THF using potassium carbonate as a base. Alkylation of piperazine with propargyl bromide under these conditions gave 4 in 14% isolated yield. Methylation of the hydroxyl group on 3a and reductive amination of the carbonyl with 4 using sodium cyanoborohydride and catalytic amounts of acetic acid in methanol gave the alkyne 5 in 55% yield (Abdel-Magid and Mehrman 2006). mBRef-1 was prepared following the same procedure used to produce 5 and resulted in a comparable yield of 58%. Compounds 5 and mBRef-1 were purified by semi-preparative RP-HPLC before being employed in Click reactions or as a blocking agent, respectively.

2-Fluoroethylazide (6) was synthesised following a previously published procedure (Ackermann et al. 2011; Glaser and Årstad 2007). Briefly, 2-fluoroethyl 4-toluenesulfonate was treated with sodium azide at room temperature for 24 h. The crude reaction mixture was used in the next step without prior purification. A copper (I) source in conjunction with 10 mol% of a reducing agent was used as a catalyst for the 1,3-dipolar cycloaddition between 5 and 6 which gave the 1,4-substituted triazole mBPET-1 in 25% yield (Hein and Fokin 2010; Meldal and Tornøe 2008). ^1^H-NMR analysis of mBPET-1 showed a singlet at 7.90 ppm corresponding to the 1,2,3-triazole proton and the characteristic fluorine splitting of the protons on the fluoroethyl group (Additional file 1: Figure S7 a). MS analysis showed a signal at *m/z* = 450.22839 which correlates to the MH^+^ signal of mBPET-1 with a mass error of − 3.5 ppm (Additional file 1: Figure S7 c).

**Fig. 3:**
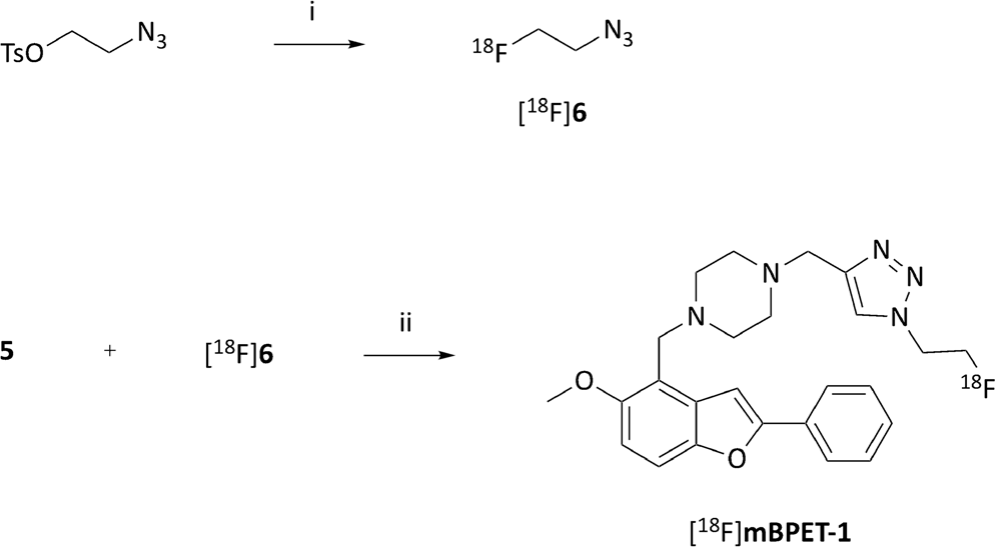
Automated radiosynthesis of [^18^F]mBPET-1. i) [K/(2.2.2)]^+^/^18^F^−^, acetonitrile, 90 °C, 7 min, 60% (n.d.c.); ii) [Cu^I^(TBTA)] PF_6_, DMF, 70 °C, 30 min; Overall RCY after 90 min: 40%

**Fig. 4:**
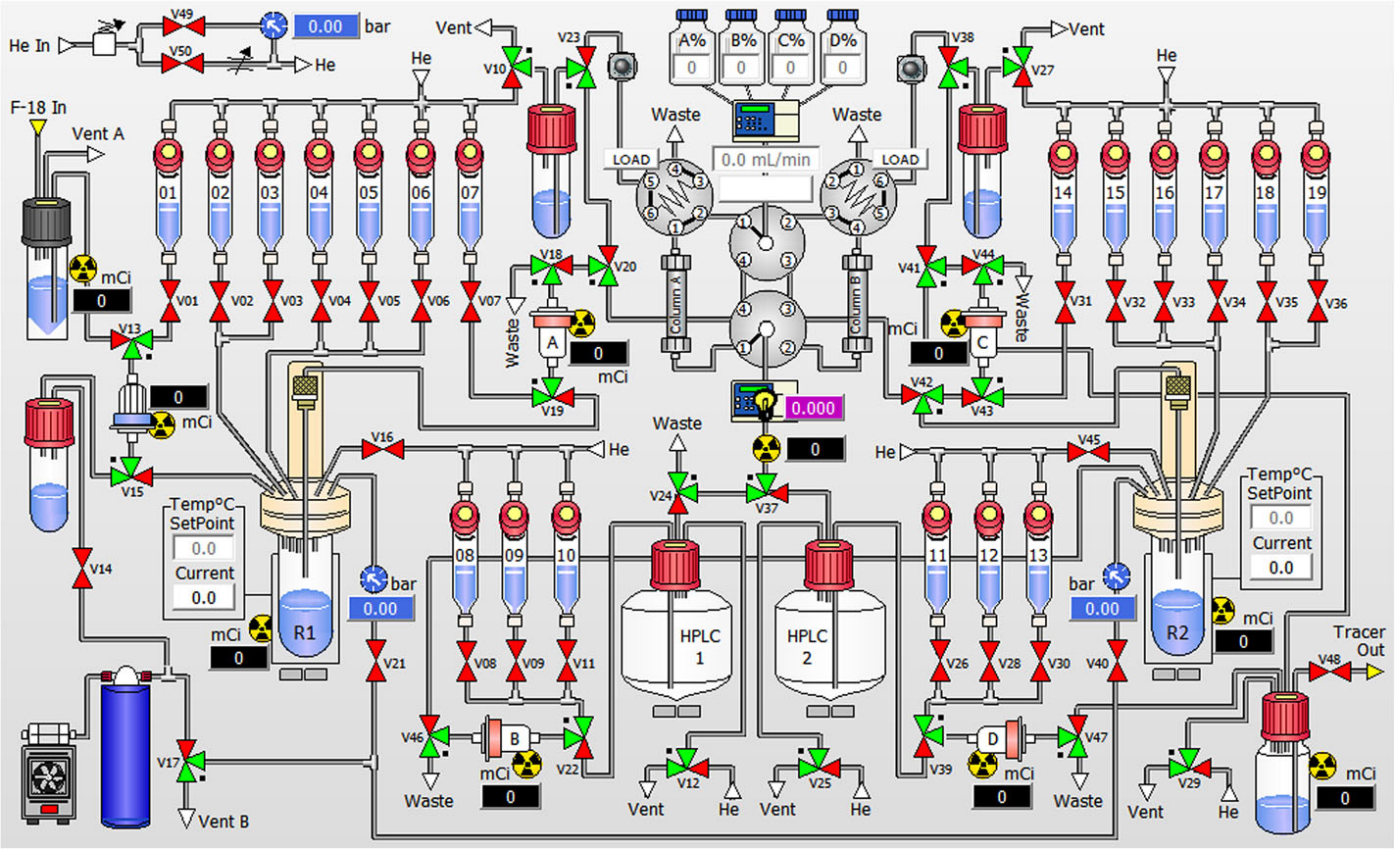
Schematic overview of iPHASE Flexlab synthesizer. Reagent setup for radiosynthesis of [^18^F]mBPET-1 is described in Table 1

Radiosynthesis of [^18^F]mBPET-1 was achieved using copper catalysed click chemistry similarly to the synthesis of the non-radioactive derivative mBPET-1 (Fig. 3). 2-Azidoethyl-4-toluenesulfonate was treated with activated n.c.a. [^18^F] fluoride to give 2-[^18^F]fluoroethylazide ([^18^F]6) which was purified by distillation. Cycloaddition of [^18^F]6 to 5 was carried out using a Cu (I)/TBTA catalyst system. All steps were performed using the iPHASE Flexlab (Melbourne, Australia) automated synthesis module (Fig. 4). The previously published procedure was modified to facilitate radiosynthesis of [^18^F]mBPET-1 (Ackermann et al. 2014).

Briefly, aqueous [^18^F]fluoride from the cyclotron target was concentrated on an ion exchange cartridge, eluted into reactor 1 (Fig. 4 - R1) with a solution of K_2_CO_3_/Kryptofix and dried azeotropically. The dry [^18^F]F^−^/cryptate was heated with 2-azidoethyl-4-toluenesulfonate and the product was subsequently isolated via distillation by heating / pressurising reactor 1 and cooling / evacuating reactor 2 (Fig. 4 - R2). Residual [^18^F]6 remaining in the tubing after the distillation was recovered by transferring a small volume of DMF from vial 7 into reactor 2. The distillate was then reacted with precursor 5 using a Cu (I)/TBTA catalyst. After evaporation of unreacted [^18^F]6 the resulting mixture was diluted with acetonitrile and water. [^18^F]mBPET-1 was isolated using HPLC-column B and collected in HPLC flask 2. The diluted product was concentrated on a C18 cartridge, washed with water and reformulated in DMSO.

**Fig. 5:**
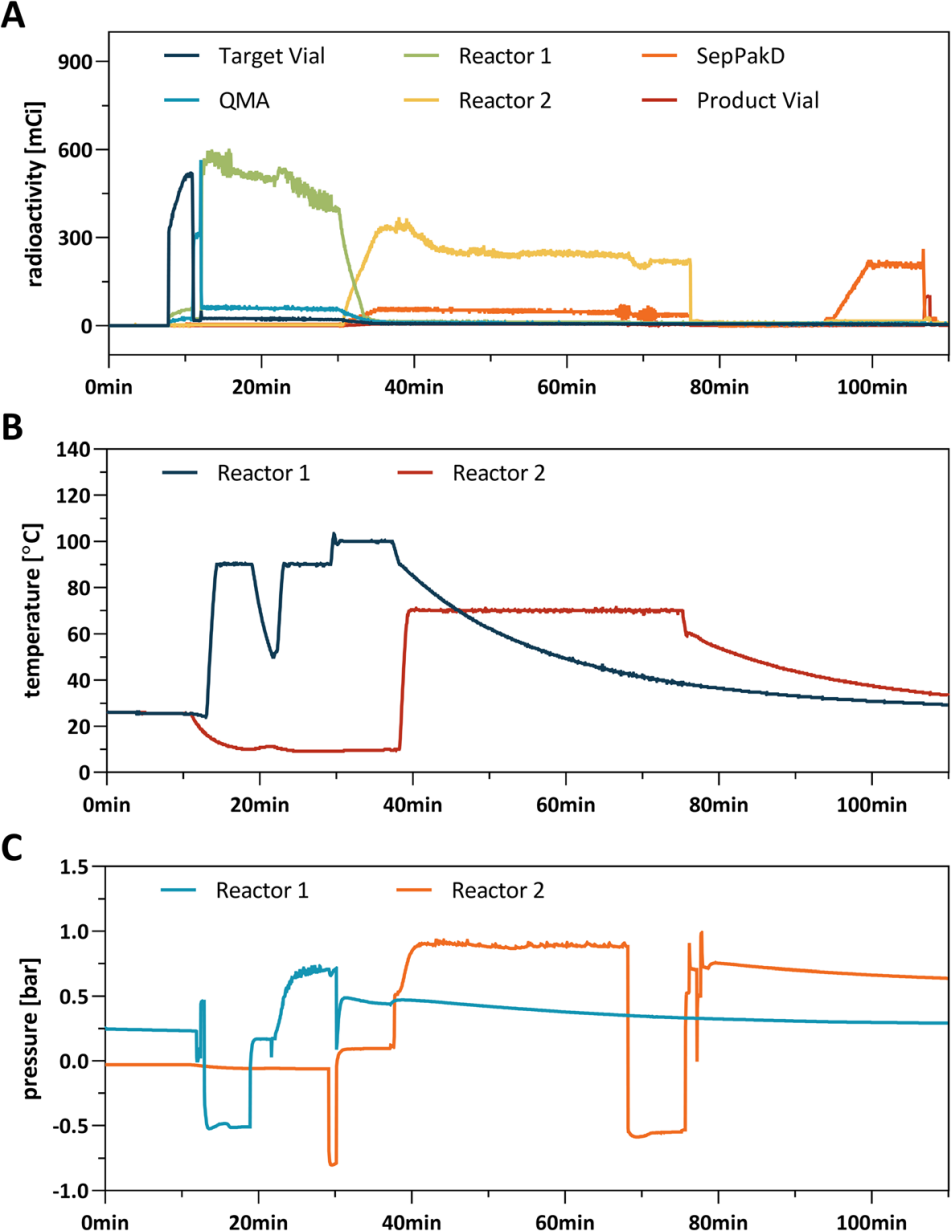
Radioactivity (**a**), temperature (**b**) and pressure (**c**) traces of the radiosynthesis of [^18^F]mBPET-1 on the iPHASE Flexlab

**Fig. 6:**
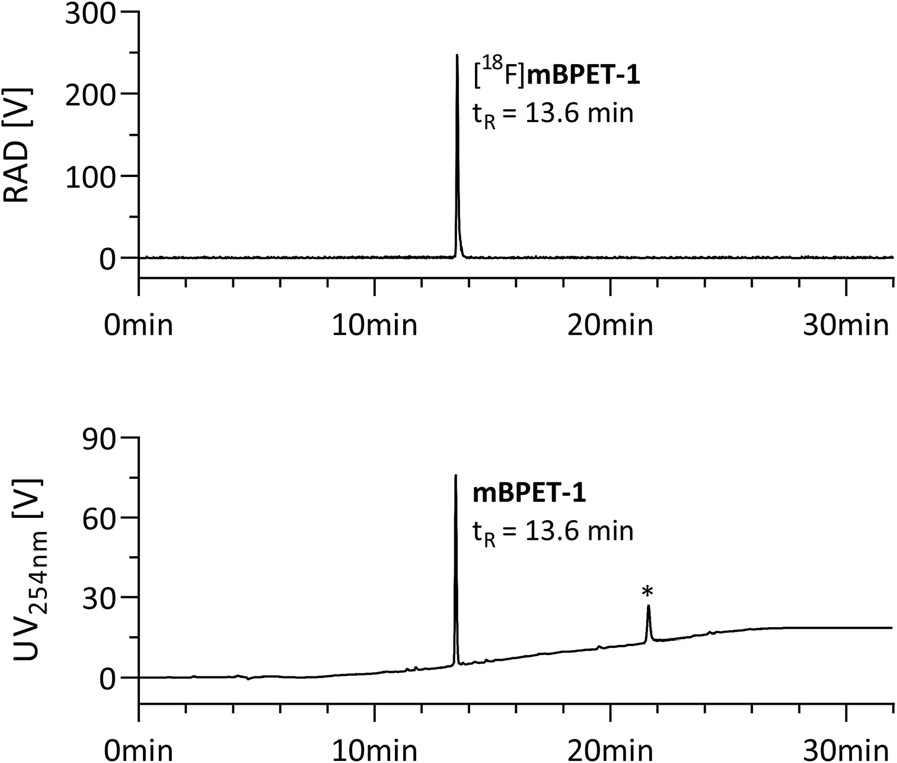
Scintillation (*top*) and UV_254nm_ (*bottom*) RP-HPLC traces of coinjected [^18^F]mBPET-1 and mBPET-1; *HPLC column contamination

Nucleophilic substitution typically afforded [^18^F]6 in 60% n.d.c. yield after distillation. The distillation process can be monitored via the radioactivity traces of R1 and R2 and should follow the trend shown in Fig. 5a. The crude triazole [^18^F]mBPET-1 was purified using semi-preparative RP-HPLC followed by reformulation using a polymer-based C18 cartridge. The overall radiochemical yield was 40% ± 5% (*n* = 6) after 90 min of synthesis. The radiochemical purity was always ≥99% at end of synthesis (EOS) and ≥ 98% after 4 h at room temperature in DMSO (Additional file 1: Table S2). The typical molar activity was 24.8 GBq/μmol (EOS) with a maximum of 78.6 GBq/μmol (EOS) produced. The logarithmic *n*-octanol/water distribution coefficient at pH 7.4 (logD_7.4_) was determined to be 0.89 (Additional file 1: Table S1). [^18^F]mBPET-1 showed high metabolic stability. Incubation with mouse S9 liver fractions at 37 °C resulted in a 0.8% drop in radiochemical purity after 3 h compared to a control (Additional file 1: Table S3). Coinjection of [^18^F]mBPET-1 and mBPET-1 confirmed the identity of the radiolabelled material (Fig. 6).

To investigate the ability of [^18^F]mBPET-1 to distinguish between RAD001 sensitive and insensitive tumour populations, breast cancer cell lines with high and low sensitivity to RAD001 were selected (Hurvitz et al. 2015b). HCC-1419 is a luminal breast cancer cell line with positive HER2 and estrogen receptor (ER) status. The reported average half maximal inhibitory concentration (IC50) when treated with RAD001 is 0.20 nM and average inhibition at 100 nM is > 100%, which classifies this cell line as RAD001 sensitive according to Hurvitz et al.. MDA-MB-468 is a basal breast cancer cell line with normal HER2 and negative ER status. The reported IC50 value when treated with RAD001 is > 100 nM with an average inhibition of 48.9% at that concentration. MDA-MB-468 is classified as a RAD001 resistant cell line, although the term insensitive would be more suitable due to the fact that these cells still show some response to RAD001 treatment.

**Fig. 7:**
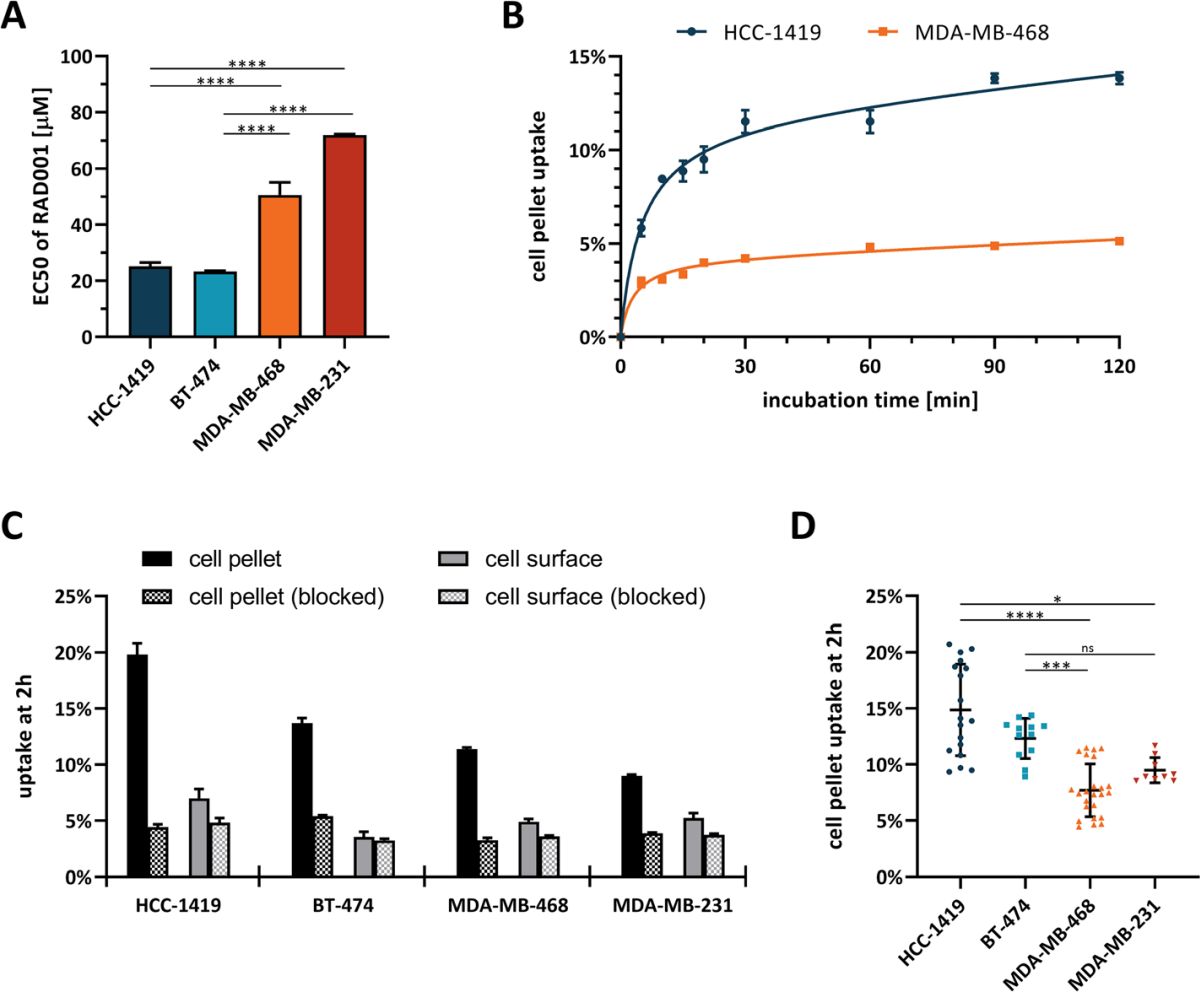
EC50 values in 4 different breast cancer cell lines treated with RAD001 for 3 days (**a**). Cell pellet uptake of [^18^F]mBPET-1 in HCC-1419 and MDA-MB-468 cells over time (**b**). Unblocked and blocked (treated with 10 μM mBRef-1) uptake of [^18^F]mBPET-1 in the cell pellet and on the cell surface of 4 different breast cancer cell lines after 2 h (**c**). Cell pellet uptake of [^18^F]mBPET-1 in 4 different breast cancer cell lines at 2 h (**d**). Data are expressed as mean ± SD

The reported RAD001 sensitivity of these cell lines was confirmed by determining the half maximal effective concentration (EC50) using the colorimetric MTS proliferation assay (Additional file 1: Table S4). Cytotoxicity of RAD001 was significantly different to the vehicle control in all cell lines at higher concentrations (Additional file 1: Figure S9). Mean EC50 values of RAD001 in HCC-1419 and BT-474 cells were 25.17 μM ± 1.14 μM and 23.27 μM ± 0.29 μM, respectively (Fig. 7a). Mean EC50 values of RAD001 in MDA-MB-468 and MDA-MB-231 cells were 50.65 μM ± 3.64 μM and 72.02 μM ± 0.26 μM, respectively. While the absolute EC50 values differ from the reported IC50 data, presumably due to the different assay used by Hurvitz et al., the overall trend of high/low RAD001 sensitivity was confirmed. Based on these results, the HCC-1419/BT-474 and MDA-MB-468/MDA-MB-231 cell lines were used as a model system for RAD001 treatment sensitivity.

Initially, dynamic cell uptake of [^18^F]mBPET-1 was investigated in vitro in HCC-1419 and MDA-MB-468 cell lines over a time frame of 120 min (Fig. 7b). Uptake in the cell pellet was used as an indicator of mTOR expression or activation levels and should correlate with the RAD001 sensitivity of each cell line. The kinetic profiles of [^18^F]mBPET-1 were similar in both cell lines reaching an uptake equilibrium after incubation for 60–90 min at 37 °C. Uptake of [^18^F]mBPET-1 in RAD001 sensitive HCC-1419 cells reached 13.8% ± 0.2% at equilibrium compared to 4.9% ± 0.1% in RAD001 insensitive MDA-MB-468 cells (Additional file 1: Table S5). The maximum ratio of the cell pellet uptake between the two cell lines was 2.83.

Uptake specificity was determined by treating cells with mBRef-1 at a concentration of 10 μM, in addition to [^18^F]mBPET-1 (Fig. 7c). mBRef-1 competes with [^18^F]mBPET-1 for the same binding sites and thereby reduces target specific binding of the latter. mBRef-1 competition reduced uptake of [^18^F]mBPET-1 in all cell lines indicating that there is target specific binding in each of them. This is not surprising considering mTOR is central to survival and a natural component of cells. Interestingly, the level of [^18^F]mBPET-1 uptake in blocked experiments seems to be similar across cell lines (Additional file 1: Table S6). This indicates a general pool of non-specific uptake. In this experiment, cell surface bound [^18^F]mBPET-1 was removed by including an acidic washing step with 50 mM acetate buffer pH 5. Blocking showed that while there is considerable binding to the cell surface (3.6% - 7.0%), this is mostly non-specific (specific binding: 0.3% - 2.2%).

Figure 7d shows a summary of [^18^F]mBPET-1 cell uptake after incubation for 2 h from multiple experiments performed in triplicate (Additional file 1: Table S7). Mean uptake in RAD001 sensitive HCC-1419 and BT-474 cell lines is 14.9% ± 4.0% (*n* = 6) and 12.3% ± 1.7% (*n* = 4), respectively. Mean uptake in RAD001 insensitive MDA-MB-468 and MDA-MB-231 cell lines is 7.7% ± 2.3% (*n* = 8) and 9.5% ± 1.1% (*n* = 3), respectively. The difference in [^18^F]mBPET-1 uptake between either of the RAD001 sensitive cell lines and MDA-MB-468 is highly significant. A weaker, but statistically significant difference between HCC-1419 and MDA-MB-231 cells is also evident.

While these preliminary experiments suggest that [^18^F]mBPET-1 may be a suitable probe to distinguish between RAD001 sensitive and insensitive cell lines, more work is required to characterise the relation between mTOR expression / activation and RAD001 sensitivity in the employed cell lines.

## Conclusion

In conclusion, we synthesized a substituted benzofuran scaffold equipped with an alkyne in 27.5% yield over 7 linear steps. Fully automated radiolabelling using Click chemistry proceeded in 40% RCY and gave [^18^F]mBPET-1 in excellent radiochemical purity and good molar activity. In vitro studies confirmed the specificity of observed cell uptake and showed 1.3–1.9-fold increased cell uptake of [^18^F]mBPET-1 in RAD001 sensitive compared to RAD001 insensitive cells across 4 breast cancer cell lines. These results warrant further studies in murine xenografts to expand on the biological properties of this molecular probe and evaluate its suitability as a predictor of RAD001 treatment response.

## Supplementary information

**Additional file 1: Figure S1a.** ^1^H-NMR of 2-Bromo-1,4-dihydroxybenzene. **Figure S2a.** ^1^H-NMR of 2-Phenylbenzofuran-5-ol. **Figure S3a.** ^1^H-NMR of 5-hydroxy-2-phenylbenzofuran-4-carbaldehyde (**3a**). **Figure S4a.** ^1^H-NMR of 5-Methoxy-2-phenylbenzofuran-4-carbaldehyde (**3b**). **Figure S5a.** ^1^H-NMR of 1-(Prop-2-yn-1-yl) piperazine. **Figure S6a.** ^1^H-NMR of 1-((5-Methoxy-2-phenylbenzofuran-4-yl) methyl)-4-(prop-2-yn-1-yl) piperazine (**5**). **Figure S7a.** ^1^H-NMR of 1-((1-(2-Fluoroethyl)-1H-1,2,3-triazol-4-yl) methyl)-4-((5-methoxy-2-phenylbenzofuran-4-yl) methyl) piperazine (**mBPET-1**). **Figure S8a.** ^1^H-NMR of 1′-((5-methoxy-2-phenylbenzofuran-4-yl) methyl)-1,4′-bipiperidine (**mBRef-1**). **Table S1.** Distribution coefficient of [^18^F]**mBPET-1** @ pH 7.4. **Table S2.** Radiochemical purity of [^18^F]**mBPET-1** incubated in DMSO at room temperature. **Table S3.** Radiochemical purity (RP) of [^18^F]**mBPET-1** incubated with or without (control) mouse S9 liver fractions at 37 °C as determined by RP-HPLC. **Table S4a.** MTS cytotoxicity assays of HCC-1419 breast cancer cells treated with either RAD001 or vehicle control (DMSO) for 3 days. **Figure S9.** Summary of MTS cytotoxicity assays in 4 different breast cancer cell lines. Experiments were performed in triplicates and repeated three times. Data are expressed as their mean ± SD. **Table S5.** Cell uptake assay of [^18^F]**mBPET-1** in HCC-1419 and MDA-MB-468 breast cancer cells from 0 to 120 min. Cell uptake expressed as the percentile of decay corrected total dose added to cells. **Table S6a.** Blocked and unblocked cell pellet uptake of [^18^F]**mBPET-1** after incubation for 2 h at 37 °C. **Table S7.** Cell pellet uptake of [^18^F]**mBPET-1** after incubation for 2 h at 37 °C. Summary of multiple experiments performed in triplicates.

## Data Availability

The datasets used and/or analysed during the current study are available from the corresponding author on reasonable request.

## Abbreviations

AKT: Protein kinase B
CDK4/6: Cyclin-dependent kinase 4/6
DEPT: Distortionless enhancement by polarization transfer
DMF: Dimethylformamide
DMSO: Dimethyl sulfoxide
EC50: Half maximal effective concentration
EOS: End of synthesis
ER+/−: Estrogen receptor positive/negative
ESI-MS: Electrospray ionization mass spectrometry
FT-ICR: Fourier-transform ion cyclotron resonance
G-6-P-DH: Glucose-6-phosphate dehydrogenase
HER2: Human epidermal growth factor receptor 2
HR+/−: Hormone receptor positive/negative
HRMS: High resolution mass spectrometry
HTS: High throughput screening
IC50: Half maximal inhibitory concentration
IHC: Immunohistochemistry
LCMS: Liquid chromatography coupled to mass spectrometry
m/z: MASS (m) to charge (z) ratio
mBPET-1: mTOR Benzofuran PET compound - derivative 1
mBRef-1: mTOR Benzofuran Reference compound - derivative 1
MeV: Megaelectronvolt
Mp: Melting point
mTOR: mammalian target of rapamycin
mTORC1: mTOR complex 1
MTS: 3-(4,5-Dimethylthiazol-2-yl)-5-(3-carboxymethoxyphenyl)-2-(4-sulfophenyl)-2*H*-tetrazolium
n.c.a.: no-carrier-added
n.d.c.: non decay-corrected
NADPH/NADP: Nicotinamide adenine dinucleotide phosphate
NMR: Nuclear magnetic resonance
P: Cell pellet fraction
PBS: Phosphate buffered saline
PDK1: Phosphoinositide-dependent kinase-1
PET: Positron emission tomography
PI3K: Phosphatidylinositide 3-kinase
pS6: phospho-S6
PTEN: Phosphatase and tensin homolog
RAD001: Everolimus
RCY: Radiochemical yield (as defined by SRS)
RP-HPLC: Reversed-phase high-performance liquid chromatography
S: Supernatant fraction
SD: Standard deviation
SPE: Solid phase extraction
SPECT: Single-photon emission computed tomography
TBTA: Tris [(1-benzyl-1H-1,2,3-triazol-4-yl) methyl] amine
THF: Tetrahydrofuran
TLC: Thin layer chromatography
TOF: Time of flight
U: Enzyme unit
UHPLC: Ultra-high performance liquid chromatography
