# Supplementary material for: Synthesis and validation of [18F]mBPET-1, a fluorine-18 labelled mTOR inhibitor derivative based on a benzofuran backbone
Source: EJNMMI Radiopharm Chem. 2020 Jan 23;5:3. doi: 10.1186/s41181-020-0089-9 (PMC6977806; doi:10.1186/s41181-020-0089-9)
Supplement: Supplementary file 1 — Additional file 1: Figure S1a. 1H-NMR of 2-Bromo-1,4-dihydroxybenzene. Figure S2a. 1H-NMR of 2-Phenylbenzofuran-5-ol. Figure S3a. 1H-NMR of 5-hydroxy-2-phenylbenzofuran-4-carbaldehyde (3a). Figure S4a. 1H-NMR of 5-Methoxy-2-phenylbenzofuran-4-carbaldehyde (3b). Figure S5a. 1H-NMR of 1-(Prop-2-yn-1-yl) piperazine. Figure S6a. 1H-NMR of 1-((5-Methoxy-2-phenylbenzofuran-4-yl) methyl)-4-(prop-2-yn-1-yl) piperazine (5). Figure S7a. 1H-NMR of 1-((1-(2-Fluoroethyl)-1H-1,2,3-triazol-4-yl) methyl)-4-((5-methoxy-2-phenylbenzofuran-4-yl) methyl) piperazine (mBPET-1). Figure S8a. 1H-NMR of 1′-((5-methoxy-2-phenylbenzofuran-4-yl) methyl)-1,4′-bipiperidine (mBRef-1). Table S1. Distribution coefficient of [18F]mBPET-1 @ pH 7.4. Table S2. Radiochemical purity of [18F]mBPET-1 incubated in DMSO at room temperature. Table S3. Radiochemical purity (RP) of [18F]mBPET-1 incubated with or without (control) mouse S9 liver fractions at 37 °C as determined by RP-HPLC. Table S4a. MTS cytotoxicity assays of HCC-1419 breast cancer cells treated with either RAD001 or vehicle control (DMSO) for 3 days. Figure S9. Summary of MTS cytotoxicity assays in 4 different breast cancer cell lines. Experiments were performed in triplicates and repeated three times. Data are expressed as their mean ± SD. Table S5. Cell uptake assay of [18F]mBPET-1 in HCC-1419 and MDA-MB-468 breast cancer cells from 0 to 120 min. Cell uptake expressed as the percentile of decay corrected total dose added to cells. Table S6a. Blocked and unblocked cell pellet uptake of [18F]mBPET-1 after incubation for 2 h at 37 °C. Table S7. Cell pellet uptake of [18F]mBPET-1 after incubation for 2 h at 37 °C. Summary of multiple experiments performed in triplicates. [file 41181_2020_89_MOESM1_ESM.docx]

**Supplementary Material**

**Synthesis and validation of [^18^F]mBPET-1, a fluorine-18 labelled mTOR inhibitor derivative based on a benzofuran backbone**

Christian W. Wichmann^1,2,3,4*^, Yit Wooi Goh^2^, Adam C. Parslow^3,4^, Angela Rigopoulos^3,4^, Nancy Guo^3^, Andrew M. Scott^1,2,3,4^, Uwe Ackermann^1,2,3,4^ and Jonathan M. White^1^.

^*^ Correspondence: [christian.wichmann@onjcri.org.au](mailto:christian.wichmann@onjcri.org.au)

1. The University of Melbourne, Parkville, VIC 3010, Australia
2. Department of Molecular Imaging and Therapy, Austin Hospital, 145 Studley Road, Heidelberg, VIC 3084, Australia
3. Olivia Newton-John Cancer Research Institute, 145 Studley Road, Heidelberg, VIC 3084, Australia
4. School of Cancer Medicine, La Trobe University, Plenty Road & Kingsbury Drive, Bundoora, VIC 3086, Australia

**Figure S1 a:** ^1^H-NMR of 2-Bromo-1,4-dihydroxybenzene (**1**).

**Figure S1 b:** ^13^C-NMR of 2-Bromo-1,4-dihydroxybenzene (**1**).

**Figure S2 a:** ^1^H-NMR of 2-Phenylbenzofuran-5-ol (**2**).

**Figure S2 b:** ^13^C-NMR of 2-Phenylbenzofuran-5-ol (**2**).

**Figure S3 a:** ^1^H-NMR of 5-hydroxy-2-phenylbenzofuran-4-carbaldehyde (**3a**).

**Figure S3 b:** ^13^C-NMR of 5-hydroxy-2-phenylbenzofuran-4-carbaldehyde (**3a**).

**Figure S3 c:** ^1^H-^1^H-gCOSY of 5-hydroxy-2-phenylbenzofuran-4-carbaldehyde (**3a**).

**Figure S4 a:** ^1^H-NMR of 5-Methoxy-2-phenylbenzofuran-4-carbaldehyde (**3b**).

**Figure S4 b:** ^13^C-NMR of 5-Methoxy-2-phenylbenzofuran-4-carbaldehyde (**3b**).

**Figure S5 a:** ^1^H-NMR of 1-(Prop-2-yn-1-yl)piperazine (**4**).

**Figure S5 b:** ^13^C-NMR of 1-(Prop-2-yn-1-yl)piperazine (**4**).

**Figure S5 c:** High-res. MS of 1-(Prop-2-yn-1-yl)piperazine (**4**).


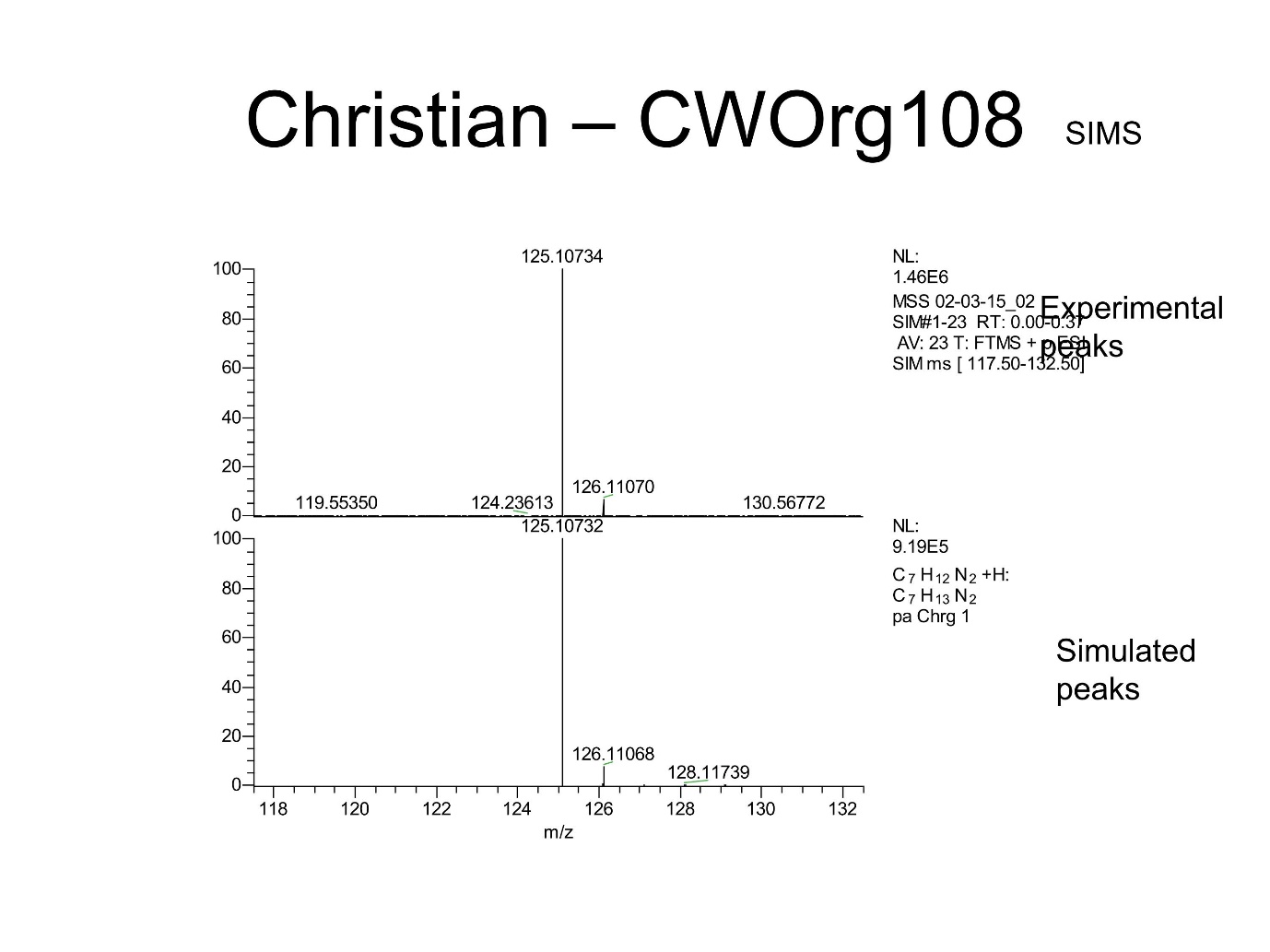


**Figure S6 a:** ^1^H-NMR of 1-((5-Methoxy-2-phenylbenzofuran-4-yl)methyl)-4-(prop-2-yn-1-yl)piperazine (**5**).

**Figure S6 b:** ^13^C-NMR of 1-((5-Methoxy-2-phenylbenzofuran-4-yl)methyl)-4-(prop-2-yn-1-yl)piperazine (**5**).

**Figure S6 c:** High-res. MS of 1-((5-Methoxy-2-phenylbenzofuran-4-yl)methyl)-4-(prop-2-yn-1-yl)piperazine (**5**).

Simulated peaks

Experimental peaks

**Figure S7 a:** ^1^H-NMR of 1-((1-(2-Fluoroethyl)-1H-1,2,3-triazol-4-yl)methyl)-4-((5-methoxy-2-phenylbenzofuran-4-yl)methyl)piperazine (**mBPET-1**).

**Figure S7 b:** ^13^C-NMR of 1-((1-(2-Fluoroethyl)-1H-1,2,3-triazol-4-yl)methyl)-4-((5-methoxy-2-phenylbenzofuran-4-yl)methyl)piperazine (**mBPET-1**).

**Figure S7 c:** High-res. MS of 1-((1-(2-Fluoroethyl)-1H-1,2,3-triazol-4-yl)methyl)-4-((5-methoxy-2-phenylbenzofuran-4-yl)methyl)piperazine (**mBPET-1**).

Simulated peaks

Experimental peaks

**Figure S8 a:** ^1^H-NMR of 1'-((5-methoxy-2-phenylbenzofuran-4-yl)methyl)-1,4'-bipiperidine (**mBRef-1**).

**Figure S8 b:** ^13^C-NMR of 1'-((5-methoxy-2-phenylbenzofuran-4-yl)methyl)-1,4'-bipiperidine (**mBRef-1**).

**Figure S8 c:** High-res. MS of 1'-((5-methoxy-2-phenylbenzofuran-4-yl)methyl)-1,4'-bipiperidine (**mBRef-1**).

Simulated peaks

Experimental peaks

**Figure S8 d:** The crystal structure of 1'-((5-methoxy-2-phenylbenzofuran-4-yl)methyl)-1,4'-bipiperidine (**mBRef-1**) has been deposited to CSD. The image below is a reproduction from the relevant publication (White et al. 2019).


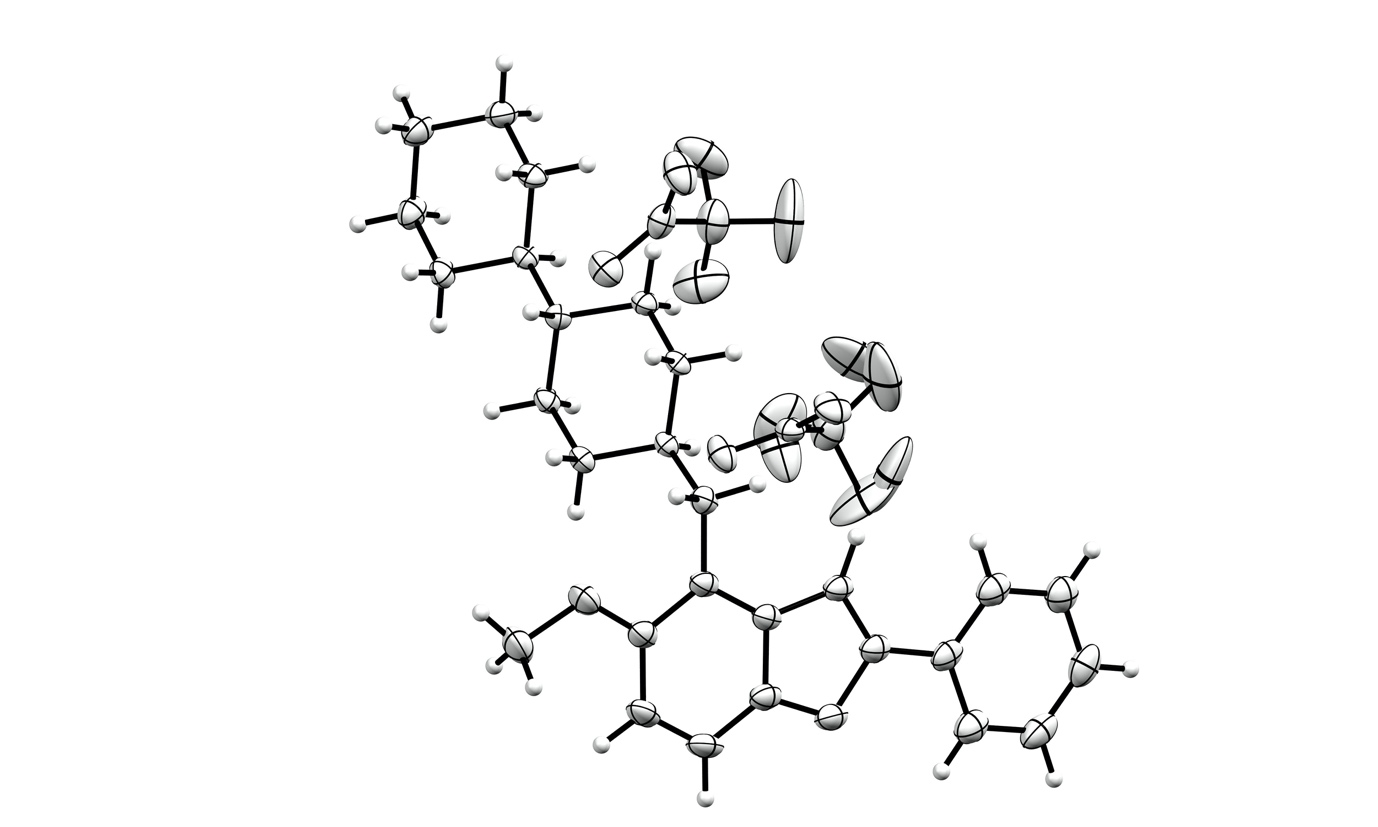


**Table S1:** Distribution coefficient of [^18^F]**mBPET-1** @ pH 7.4.

| Replicate | cpm (n-Octanol) | cpm (Phosphate Buffer) | Partition coefficient D_7.4_ | log D_7.4_ |
| --- | --- | --- | --- | --- |
| 1 | 862016.2 | 112161.7 | 7.685 | 0.89 |
| 2 | 843620.4 | 111176.4 | 7.588 | 0.88 |
| 3 | 856717.4 | 110475.9 | 7.755 | 0.89 |

**Table S2:** Radiochemical purity of [^18^F]**mBPET-1** incubated in DMSO at room temperature.

| time [h] | Radiochemical purity (HPLC) [%] |
| --- | --- |
| 0 | 99.1309 |
| 1 | 99.3466 |
| 2 | 99.0621 |
| 3 | 98.7593 |
| 4 | 98.3037 |

**Table S3:** Radiochemical purity (RP) of [^18^F]**mBPET-1** incubated with or without (control) mouse S9 liver fractions at 37°C as determined by RP-HPLC.

| time [min] | RP of [^18^F]**mBPET-1** with mouse S9 (HPLC) [%] | RP of [^18^F]**mBPET-1** control (HPLC) [%] |
| --- | --- | --- |
| 0 | 98.5711 | 98.5711 |
| 30 | 96.9903 |  |
| 60 | 96.8903 |  |
| 90 | 96.7329 |  |
| 180 | 96.2272 | 97.0489 |

**Table S4 a:** MTS cytotoxicity assays of HCC-1419 breast cancer cells treated with either RAD001 or vehicle control (DMSO) for 3 days. Cell viability expressed as percentile of untreated control wells. Three replicates performed in triplicates.

| **concentration (µM)** | **RAD001 treated cell viability [%]** | | | **DMSO treated cell viability [%]** | | |
| --- | --- | --- | --- | --- | --- | --- |
| Replicate 1 | | | | | | |
| 100 | 10.135 | 12.261 | 10.985 | 42.276 | 44.183 | 47.235 |
| 75 | 9.709 | 10.985 | 10.56 | 53.338 | 49.142 | 55.626 |
| 56.25 | 10.985 | 11.836 | 10.985 | 60.203 | 63.255 | 62.111 |
| 42.1875 | 11.836 | 11.41 | 10.56 | 66.306 | 64.781 | 64.399 |
| 31.64063 | 9.284 | 36.074 | 36.499 | 71.265 | 73.554 | 68.214 |
| 23.73047 | 33.948 | 49.256 | 65.415 | 54.863 | 76.224 | 74.698 |
| 17.79785 | 64.139 | 73.069 | 81.148 | 83.471 | 87.285 | 79.657 |
| 13.34839 | 72.218 | 76.045 | 75.62 | 77.75 | 81.182 | 76.605 |
| 10.01129 | 67.966 | 72.218 | 69.667 | 78.512 | 81.945 | 81.564 |
| 7.508469 | *197.661** | 57.335 | 63.714 | 83.09 | 76.224 | 82.708 |
| Replicate 2 | | | | | | |
| 100 | 10.731 | 9.316 | 10.024 | 56.368 | 51.769 | 56.722 |
| 75 | 10.377 | 10.377 | 11.085 | 59.198 | 59.552 | 56.368 |
| 56.25 | 9.67 | 9.67 | 11.792 | 63.797 | 71.226 | 84.67 |
| 42.1875 | 9.67 | 10.377 | 12.5 | 77.241 | 72.642 | 93.514 |
| 31.64063 | 10.377 | 10.731 | 25.59 | 84.316 | 72.288 | 85.377 |
| 23.73047 | 31.25 | 45.047 | 42.925 | 77.241 | 76.887 | 90.33 |
| 17.79785 | 53.184 | 65.212 | 63.09 | 78.656 | 79.009 | 97.406 |
| 13.34839 | 68.042 | 72.642 | 73.703 | 75.825 | 76.179 | *121.816** |
| 10.01129 | 65.212 | 65.212 | 62.736 | 87.854 | 82.901 | *131.014** |
| 7.508469 | 60.967 | 56.368 | 58.491 | 80.778 | 81.486 | *127.476** |
| Replicate 3 | | | | | | |
| 100 | 8.925 | 10.335 | 10.687 | 59.659 | 60.716 | 55.784 |
| 75 | 9.63 | 10.335 | 12.096 | 67.763 | 66.706 | 64.592 |
| 56.25 | 9.63 | 10.687 | 11.744 | 76.218 | 76.923 | 82.913 |
| 42.1875 | 9.63 | 10.687 | 12.801 | 89.959 | 79.037 | 86.436 |
| 31.64063 | 9.982 | 13.506 | 18.438 | 102.995 | 89.254 | 96.653 |
| 23.73047 | 44.51 | 51.204 | 49.794 | 99.119 | 89.254 | 92.777 |
| 17.79785 | 67.763 | 70.229 | 79.037 | 95.244 | 100.176 | 103.347 |
| 13.34839 | 86.788 | 76.923 | 78.685 | 104.404 | 93.834 | *123.077** |
| 10.01129 | 76.218 | 73.752 | 71.286 | 99.472 | 94.539 | *130.476** |
| 7.508469 | 70.581 | 66.353 | 67.058 | 94.539 | 94.891 | *117.792** |

**Table S4 b:** MTS cytotoxicity assays of BT-474 breast cancer cells treated with either RAD001 or vehicle control (DMSO) for 3 days. Cell viability expressed as percentile of untreated control wells. Three replicates performed in triplicates.

| **concentration (µM)** | **RAD001 treated cell viability [%]** | | | **DMSO treated cell viability [%]** | | |
| --- | --- | --- | --- | --- | --- | --- |
| Replicate 1 | | | | | | |
| 100 | 10.808 | 10.451 | 11.164 | 74.941 | 92.755 | 134.086 |
| 75 | 9.739 | 11.164 | 14.371 | 75.653 | 93.468 | 107.72 |
| 56.25 | 10.808 | 11.164 | 14.014 | 79.216 | 99.881 | 125.534 |
| 42.1875 | 10.095 | 11.164 | 14.371 | 93.468 | 95.606 | 121.971 |
| 31.64063 | 9.739 | 9.739 | 14.371 | 92.755 | 111.283 | 141.924 |
| 23.73047 | 13.658 | 36.817 | 39.667 | 91.686 | 123.397 | 157.957 |
| 17.79785 | 86.342 | 86.342 | 87.055 | 80.285 | 120.546 | 192.162 |
| 13.34839 | 86.698 | 87.411 | 89.549 | 87.767 | 170.071 | 208.907 |
| 10.01129 | 76.01 | 72.09 | 71.021 | 117.696 | 170.428 | 191.805 |
| 7.508469 | 64.252 | 59.62 | 59.976 | 147.268 | 179.691 | 73.159 |
| Replicate 2 | | | | | | |
| 100 | 11.874 | 12.303 | 13.162 | 95.565 | 108.87 | 133.333 |
| 75 | 10.587 | 11.016 | 16.595 | 101.144 | 110.587 | 131.187 |
| 56.25 | 12.303 | 13.591 | 16.595 | 98.14 | 119.599 | 132.475 |
| 42.1875 | 12.303 | 14.449 | 17.454 | 109.728 | 110.587 | 142.775 |
| 31.64063 | 12.732 | 12.732 | 17.024 | 117.883 | 132.046 | 150.501 |
| 23.73047 | 24.32 | 28.612 | 20.458 | 108.87 | 137.625 | 165.522 |
| 17.79785 | 99.428 | 98.569 | 121.745 | 98.999 | 150.501 | 190.415 |
| 13.34839 | 103.72 | 108.441 | 112.303 | 99.857 | 192.99 | 231.617 |
| 10.01129 | 86.981 | 88.698 | 92.561 | 112.303 | 217.454 | 221.316 |
| 7.508469 | 80.973 | 82.69 | 79.685 | 141.488 | 216.595 | 212.303 |
| Replicate 3 | | | | | | |
| 100 | 12.46 | 9.585 | 13.419 | 97.284 | 98.243 | 91.534 |
| 75 | 14.377 | 7.668 | 13.898 | 99.201 | 104.952 | 92.971 |
| 56.25 | 12.46 | 14.377 | 11.981 | 98.722 | 107.348 | 95.847 |
| 42.1875 | 14.856 | 11.022 | 14.856 | 101.597 | 105.911 | 96.326 |
| 31.64063 | 13.419 | 11.502 | 12.46 | 108.307 | 108.786 | 94.409 |
| 23.73047 | 52.236 | 57.029 | 48.882 | 103.035 | 119.808 | 92.971 |
| 17.79785 | 120.288 | 99.201 | 118.85 | 108.786 | 114.537 | 107.827 |
| 13.34839 | 119.329 | 114.537 | 132.268 | 107.348 | 113.099 | 113.099 |
| 10.01129 | 93.45 | 92.492 | 94.409 | 93.45 | 105.911 | 147.125 |
| 7.508469 | 93.93 | 85.783 | 92.492 | 92.492 | 154.313 | *197.444** |

**Table S4 c:** MTS cytotoxicity assays of MDA-MB-468 breast cancer cells treated with either RAD001 or vehicle control (DMSO) for 3 days. Cell viability expressed as percentile of untreated control wells. Three replicates performed in triplicates.

| **concentration (µM)** | **RAD001 treated cell viability [%]** | | | **DMSO treated cell viability [%]** | | |
| --- | --- | --- | --- | --- | --- | --- |
| Replicate 1 | | | | | | |
| 100 | 8.582 | 9.312 | 9.312 | 95.496 | 110.103 | 111.199 |
| 75 | 9.312 | 8.947 | 9.677 | 91.844 | 101.704 | 109.008 |
| 56.25 | 8.582 | 17.346 | 23.554 | 92.575 | 94.4 | 96.226 |
| 42.1875 | 53.5 | 75.046 | 88.192 | 87.827 | 98.418 | 106.086 |
| 31.64063 | 90.383 | 107.182 | 103.165 | 93.67 | 83.81 | 96.957 |
| 23.73047 | 101.339 | 107.182 | 102.8 | 86.732 | 88.192 | 91.114 |
| 17.79785 | 102.069 | 93.67 | 97.322 | 87.462 | 90.018 | 99.513 |
| 13.34839 | 90.383 | 92.575 | 91.114 | 90.749 | 94.035 | 101.704 |
| 10.01129 | 84.906 | 87.827 | 88.192 | 84.906 | 94.766 | 104.991 |
| 7.508469 | 85.271 | 83.08 | 84.54 | 87.827 | 96.592 | 103.53 |
| Replicate 2 | | | | | | |
| 100 | 7.893 | 7.597 | 7.301 | 95.807 | 99.063 | 107.943 |
| 75 | 7.005 | 7.893 | 9.669 | 90.479 | 110.607 | 116.823 |
| 56.25 | 31.87 | 34.534 | 44.894 | 96.991 | 109.127 | 112.679 |
| 42.1875 | 68.574 | 76.27 | 77.158 | 97.583 | 111.199 | 117.119 |
| 31.64063 | 89.295 | 90.183 | 92.551 | 103.207 | 110.607 | 106.759 |
| 23.73047 | 98.175 | 102.023 | 105.871 | 96.399 | 111.495 | 107.351 |
| 17.79785 | 97.583 | 92.551 | 96.399 | 94.919 | 107.351 | 104.391 |
| 13.34839 | 92.847 | 93.735 | 90.775 | 98.471 | 110.311 | 107.943 |
| 10.01129 | 93.143 | 91.959 | 90.479 | 99.655 | 110.015 | 111.495 |
| 7.508469 | 94.327 | 89.591 | 87.815 | 103.799 | 112.679 | 117.711 |
| Replicate 3 | | | | | | |
| 100 | 8.036 | 7.132 | 6.831 | 91.512 | 110.799 | 111.703 |
| 75 | 8.94 | 8.639 | 8.639 | 93.019 | 110.196 | 111.1 |
| 56.25 | 69.513 | 39.377 | 47.815 | 92.115 | 103.566 | 119.237 |
| 42.1875 | 74.636 | 74.636 | 77.649 | 92.416 | 110.799 | 119.839 |
| 31.64063 | 93.32 | 90.608 | 91.21 | 103.867 | 121.346 | 111.401 |
| 23.73047 | 106.278 | 105.676 | 110.497 | 107.182 | 117.127 | 113.209 |
| 17.79785 | 106.881 | 96.936 | 105.374 | 105.374 | 116.223 | 113.209 |
| 13.34839 | 103.867 | 98.443 | 96.032 | 101.758 | 113.812 | 107.785 |
| 10.01129 | 98.443 | 98.142 | 92.115 | 101.155 | 111.401 | 118.031 |
| 7.508469 | 95.128 | 92.416 | 92.416 | 106.278 | 106.278 | 116.524 |

**Table S4 d:** MTS cytotoxicity assays of MDA-MB-231 breast cancer cells treated with either RAD001 or vehicle control (DMSO) for 3 days. Cell viability expressed as percentile of untreated control wells. Three replicates performed in triplicates.

| **concentration (µM)** | **RAD001 treated cell viability [%]** | | | **DMSO treated cell viability [%]** | | |
| --- | --- | --- | --- | --- | --- | --- |
| Replicate 1 | | | | | | |
| 100 | 4.691 | 4.691 | 4.914 | 94.49 | 118.838 | 117.722 |
| 75 | 5.808 | 6.031 | 6.701 | 94.043 | 121.519 | 114.818 |
| 56.25 | 118.392 | 114.371 | 119.955 | 104.542 | 116.605 | 111.243 |
| 42.1875 | 110.573 | 106.329 | 109.903 | 120.402 | 114.147 | 113.031 |
| 31.64063 | 94.937 | 96.5 | 99.181 | 120.625 | 109.68 | 109.903 |
| 23.73047 | 90.692 | 94.267 | 100.745 | 120.402 | 108.786 | 111.914 |
| 17.79785 | 85.108 | 90.916 | 94.267 | 125.54 | 121.519 | 112.137 |
| 13.34839 | 82.651 | 83.321 | 84.214 | 118.392 | 114.594 | 114.594 |
| 10.01129 | 79.077 | 77.736 | 80.417 | 111.467 | 111.467 | 111.02 |
| 7.508469 | 79.523 | 83.098 | 80.194 | 110.573 | 113.701 | 108.786 |
| Replicate 2 | | | | | | |
| 100 | 3.681 | 4.137 | 4.364 | 89.526 | 114.801 | 120.266 |
| 75 | 8.008 | 5.275 | 7.324 | 85.199 | 111.157 | 115.028 |
| 56.25 | 119.127 | 108.425 | 116.622 | 87.476 | 107.059 | 115.939 |
| 42.1875 | 108.425 | 106.603 | 105.693 | 92.258 | 112.068 | 114.345 |
| 31.64063 | 101.594 | 98.178 | 95.674 | 104.782 | 112.068 | 113.662 |
| 23.73047 | 89.07 | 89.981 | 87.704 | 111.385 | 112.751 | 116.395 |
| 17.79785 | 84.516 | 85.655 | 85.427 | 111.157 | 110.019 | 112.751 |
| 13.34839 | 84.288 | 87.704 | 82.922 | 111.385 | 111.157 | 115.939 |
| 10.01129 | 85.427 | 79.734 | 83.15 | 118.216 | 114.118 | 115.939 |
| 7.508469 | 78.14 | 85.199 | 79.279 | 112.296 | 111.385 | 117.761 |
| Replicate 3 | | | | | | |
| 100 | 5.79 | 5.534 | 5.534 | 93.146 | 110.515 | 131.716 |
| 75 | 15.751 | 13.197 | 15.241 | 94.934 | 93.657 | 126.096 |
| 56.25 | 129.928 | 130.438 | 129.161 | 97.233 | 98.255 | 126.352 |
| 42.1875 | 119.455 | 121.243 | 115.624 | 97.999 | 127.118 | 130.183 |
| 31.64063 | 110.26 | 106.939 | 108.472 | 107.961 | 124.564 | 127.629 |
| 23.73047 | 103.874 | 102.597 | 101.831 | 117.923 | 121.499 | 123.542 |
| 17.79785 | 98.765 | 96.211 | 94.934 | 123.542 | 124.053 | 128.906 |
| 13.34839 | 100.298 | 96.722 | 96.977 | 129.161 | 122.009 | 127.118 |
| 10.01129 | 85.994 | 93.146 | 92.124 | 125.33 | 125.841 | 131.971 |
| 7.508469 | 90.081 | 94.168 | 96.722 | 124.053 | 131.205 | 128.906 |

**Figure S9:** Summary of MTS cytotoxicity assays in 4 different breast cancer cell lines. Experiments were performed in triplicates and repeated three times. Data are expressed as their mean ± SD.











**Table S5:** Cell uptake assay of [^18^F]**mBPET-1** in HCC-1419 and MDA-MB-468 breast cancer cells from 0-120min. Cell uptake expressed as the percentile of decay corrected total dose added to cells.

| **time [min]** | **Cell uptake in HCC-1419 [%]** | | | **Cell uptake in MDA-MB-468 [%]** | | |
| --- | --- | --- | --- | --- | --- | --- |
| 0 | 0 | 0 | 0 | 0 | 0 | 0 |
| 5 | 5.62 | 5.54 | 6.34 | 2.66 | 3.18 | 2.93 |
| 10 | 8.36 | 8.33 | 8.72 | 3.11 | 3.16 | 2.96 |
| 15 | 8.87 | 9.44 | 8.33 | 3.22 | 3.27 | 3.59 |
| 20 | 8.71 | 9.86 | 9.96 | 3.93 | 4.05 | 3.96 |
| 30 | 10.96 | 12.17 | 11.41 | 4.23 | 4.07 | 4.31 |
| 60 | 10.96 | 12.17 | 11.41 | 4.99 | 4.85 | 4.6 |
| 90 | 13.67 | 14.12 | 13.7 | 4.76 | 5.01 | 4.89 |
| 120 | 13.5 | 13.88 | 14.12 | 5.26 | 5.2 | 4.94 |

**Table S6 a:** Blocked and unblocked cell pellet uptake of [^18^F]**mBPET-1** after incubation for 2 hours at 37°C.

| **cell line** | **cell pellet uptake [%]** | | | **cell pellet uptake (blocked) [%]** | | |
| --- | --- | --- | --- | --- | --- | --- |
| HCC-1419 | 20.71 | 18.71 | 20.02 | 4.51 | 4.15 | 4.64 |
| BT-474 | 14.21 | 13.33 | 13.53 | 5.49 | 5.35 | 5.4 |
| MDA-MB-468 | 11.2 | 11.49 | 11.44 | 3.42 | 3.35 | 3.02 |
| MDA-MB-231 | 8.93 | 9.14 | 8.92 | 3.88 | 3.95 | 3.84 |

**Table S6 b:** Blocked and unblocked cell surface uptake of [^18^F]**mBPET-1** after incubation for 2 hours at 37°C.

| **cell line** | **cell surface uptake [%]** | | | **cell surface uptake (blocked) [%]** | | |
| --- | --- | --- | --- | --- | --- | --- |
| HCC-1419 | 6.17 | 7.83 | 6.99 | 5.3 | 4.54 | 4.62 |
| BT-474 | 3.02 | 3.69 | 3.93 | 3.26 | 3.36 | 3.08 |
| MDA-MB-468 | 4.71 | 4.85 | 5.18 | 3.53 | 3.58 | 3.69 |
| MDA-MB-231 | 4.76 | 5.31 | 5.64 | 3.81 | 3.8 | 3.63 |

**Table S7:** Cell pellet uptake of [^18^F]**mBPET-1** after incubation for 2 hours at 37°C. Summary of multiple experiments performed in triplicates.

| **HCC-1419** | **BT-474** | **MDA-MB-468** | **MDA-MB-231** |
| --- | --- | --- | --- |
| 20.71 | 14.21 | 11.2 | 8.93 |
| 18.71 | 13.33 | 11.49 | 9.14 |
| 20.02 | 13.53 | 11.44 | 8.92 |
| 20.31 | 13.22 | 11.35 | 8.71 |
| 17.93 | 13.43 | 10.9 | 8.55 |
| 15.72 | 11.26 | 10.74 | 8.55 |
| 18.57 | 14.39 | 7.4 | 9.96 |
| 19.25 | 12.62 | 7.34 | 11.67 |
| 12.39 | 12.68 | 7.33 | 10.94 |
| 11.23 | 10.86 | 7.9 |  |
| 11.79 | 9.48 | 8.08 |  |
| 10.82 | 8.92 | 8.06 |  |
| 13.5 |  | 7.76 |  |
| 13.88 |  | 7.59 |  |
| 14.12 |  | 6.77 |  |
| 9.7 |  | 7.49 |  |
| 9.48 |  | 6.26 |  |
| 9.35 |  | 6.36 |  |
|  |  | 5.26 |  |
|  |  | 5.2 |  |
|  |  | 4.94 |  |
|  |  | 4.44 |  |
|  |  | 4.69 |  |
|  |  | 4.63 |  |

**References**

White JM, Wichmann C, Ackermann U. CCDC 1959518: Experimental Crystal Structure Determination. The Cambridge Structural Database. 2019;10.5517/ccdc.csd.cc23s187.
